# Injectable reactive oxygen and nitrogen species-controlling hydrogels for tissue regeneration: current status and future perspectives

**DOI:** 10.1093/rb/rbac069

**Published:** 2022-09-20

**Authors:** Phuong Le Thi, Dieu Linh Tran, Thai Thanh Hoang Thi, Yunki Lee, Ki Dong Park

**Affiliations:** Institute of Applied Materials Science, Vietnam Academy of Science and Technology, Ho Chi Minh City 700000, Vietnam; Institute of Applied Materials Science, Vietnam Academy of Science and Technology, Ho Chi Minh City 700000, Vietnam; Department of Orthopaedics, Emory University School of Medicine, Atlanta, GA 30329, USA; Department of Orthopaedics, Emory University School of Medicine, Atlanta, GA 30329, USA; Department of Molecular Science and Technology, Ajou University, Suwon 443-749, Republic of Korea

**Keywords:** reactive oxygen/nitrogen species, oxidative stress-related diseases, injectable hydrogels, reactive oxygen/nitrogen species-scavenging/releasing/responsive properties, tissue regeneration

## Abstract

The dual role of reactive oxygen and nitrogen species (RONS) in physiological and pathological processes in biological systems has been widely reported. It has been recently suggested that the regulation of RONS levels under physiological and pathological conditions is a potential therapy to promote health and treat diseases, respectively. Injectable hydrogels have been emerging as promising biomaterials for RONS-related biomedical applications owing to their excellent biocompatibility, three-dimensional and extracellular matrix-mimicking structures, tunable properties and easy functionalization. These hydrogels have been developed as advanced injectable platforms for locally generating or scavenging RONS, depending on the specific conditions of the target disease. In this review article, the design principles and mechanism by which RONS are generated/scavenged from hydrogels are outlined alongside a discussion of their *in vitro* and *in vivo* evaluations. Additionally, we highlight the advantages and recent developments of these injectable RONS-controlling hydrogels for regenerative medicines and tissue engineering applications.

## Graphical Abstract

**Figure:**
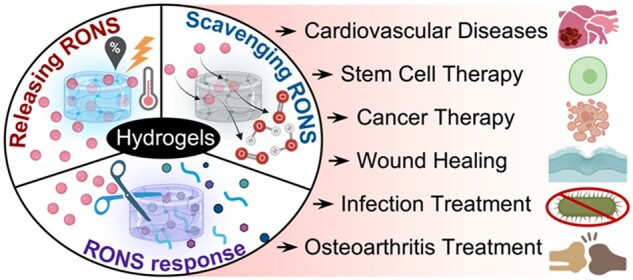

## Introduction

Reactive species or free radicals, including reactive oxygen and nitrogen species (RONS) are highly reactive molecules generated in biological systems as metabolic byproducts, as well as cellular signaling molecules [1, 2]. The term reactive oxygen species (ROS) refers to a variety of chemically derived oxygen molecules, including free radicals, such as superoxide ($O_{2}^{\cdot }$), hydroxyl (^⋅^OH), peroxyl (${\text{RO}}_{2}^{\cdot }$) and alkoxyl (RO), as well as non-radicals, such as hypochlorous acid (HOCl), ozone (O_3_), singlet oxygen (^1^O_2_) and hydrogen peroxide (H_2_O_2_) [3]. Among these ROS, H_2_O_2_ is a stable non-radical oxidant that functions as an intracellular signaling molecule that regulates kinase-driven pathways at lower physiological levels [4]. The intracellular sources of ROS include the mitochondria, endoplasmic reticulum, nuclei, plasma membranes, peroxisomes and even extracellular spaces. A number of cellular enzymes catalyze the release of ROS, such as NO synthase, peroxidases, NADPH oxidase, NADPH oxidase isoforms and glucose oxidase [5]. In contrast, nitric oxide (NO) is the primary reactive nitrogen species (RNS), produced from L-arginine by the enzyme nitric oxide synthase (NOS). There are three types of NOS in the mammalian body: endothelial NOS (eNOS or NOS3), neuronal NOS (nNOS or NOS1) and inducible NOS. In the body, a predominant amount of NO is produced from endothelial cells (ECs) at a rate of ∼0.5–4.0 × 10^−10^ mol cm^−2^ min^−1^ [6]. The other reactive form of RNS is peroxynitrite (ONOO^−^), which is formed by the interaction between ^.^NO and $O_{2}^{-}$ at low oxygen tension [6, 7]. These RONS play important physiological and pathological roles in both health and disease [8, 9]. In the past, high RONS levels have long been indicated as oxidative stress and closely related to development of numerous diseases, such as Parkinson’s and Alzheimer’s diseases, cardiovascular diseases, cancer, diabetes and rheumatoid arthritis [10, 11]. However, at the physiological level, RONS function as an important redox messenger for intracellular signaling cascades [12, 13]. For example, in the wound healing process, they regulate collagen formation, cell proliferation, differentiation and migration. RONS also has cardioprotective roles by regulating blood pressure tone, inhibiting platelet adhesion and activation and preventing smooth muscle cell proliferation. In the innate immune system, RONS are used to defend the host against pathogens [14, 15]. Therefore, understanding the mechanism of RONS action and precisely controlling their levels in biological environments would provide novel and promising therapies to treat various RONS-related diseases. Until now, many researchers have endeavored to develop RONS-scavenging/generating drug delivery systems for specific therapeutic purposes.

Hydrogels are physically or chemically crosslinked polymeric networks and they have been extensively used as advanced biomaterials for a wide range of biomedical applications, such as wound treatment, tissue regeneration and drug delivery systems [16–19]. Due to their high water content, biocompatibility, porous structure and mechanical properties similar to native tissue microenvironment, hydrogels can be used alone or loaded with various therapeutic agents (e.g. small/macromolecular drugs, cells, genes and peptides) for efficient treatment [20]. Particularly, *in situ* crosslinkable hydrogels have attracted substantial attention as powerful candidates for local/systemic drug delivery and tissue regenerative applications. The outstanding advantage of injectable hydrogels is that they can be easily delivered to various sites/organs of the body with minimal discomfort through a needle, aerosol or arthroscopic instruments, thereby preventing the incision surgery [21]. Therefore, injectable hydrogels have tremendous potential in clinical applications that gives less trauma, less blood loss, shorter surgeries and rapid recovery to the patients, compared to the traditional pre-formed hydrogels [22]. In addition, in the circumstances of the fast-growing population over the world, injectable hydrogels are attractive therapy for improving home healthcare systems, which can reduce the hospital overload induced by conventional surgeries. Depending on the specific requirements of the pathological conditions, hydrogels can be prepared from various sources, including natural polymers (e.g. collagen, gelatin, fibrin, alginate, chitosan, hyaluronic acid (HA) and dextran), synthetic polymers (e.g. ethylene oxide, vinyl alcohol or acrylic acid) or hybrid natural-synthetic polymers. Recently, various classes of injectable hydrogels have been developed as carriers to release RONS-producing/scavenging agents and have shown promising therapeutic efficacy in the prevention of RONS homeostasis-related diseases [23–26].

This review aims to summarize and evaluate the potential of injectable hydrogels as vehicles for releasing and scavenging RONS. First, based on the oxidative stress injuries induced by excess RONS in pathological conditions, recent developments in injectable ROS scavenging hydrogels have been comprehensively introduced. To exploit the unbalanced RONS levels under pathological conditions, RONS-responsive hydrogels for developing target-specific drug delivery systems have also received special attention. Second, from the viewpoint of the positive effects of RONS on cellular signaling and proliferation, we comprehensively discuss the principles and *in vitro/in vivo* evaluations of various RONS-releasing hydrogel systems. Finally, we conclude the status and future perspectives of injectable RONS-controlling hydrogels in these areas.

## ROS generation and its pathogenic roles in oxidative stress-related diseases

ROS are generated from various cellular metabolic activities, such as mitochondria, transition metal ions and peroxisome activities [27]. Among these, mitochondria is the primary source of endogenous ROS, which contributes to ∼90% of cellular ROS production, due to its main role in oxidative ATP production [28]. The most abundant mitochondrial ROS are superoxide anions, which are produced at different sites in the mitochondria, including complex I (sites IQ and IF), complex III (site IIIQo), glycerol 3-phosphate dehydrogenase, Q oxidoreductase, pyruvate dehydrogenase and 2-oxoglutarate dehydrogenase. The other ROS, such as hydrogen peroxide (H_2_O_2_), hydroxyl ions (OH-), can be converted from superoxide anions via manganese superoxide dismutase (Mn-SOD), Cu and Zn-SOD or a Fenton reaction, in the intermembrane mitochondrial space or cytosol. The other site producing ROS in the mitochondria is the cytochrome (CYP) catalytic cycle. These enzymes metabolize a wide range of substrates, such as lipids, steroid hormones and xenobiotics, to produce superoxide anions and H_2_O_2_. To protect the cell from oxidative damage of overproduction ROS, the mammalian mitochondria have various enzymatic antioxidants and non-enzymatic antioxidants, including glutathione peroxidases (GPXs), thioredoxin peroxidases, superoxide dismutases (SODs), catalase (CAT), peroxiredoxins, glutathione, thioredoxin 2, glutaredoxin 2, cytochrome c oxidase (complex IV), coenzyme Q, ascorbic acid, tocopherol, vitamin E and carotene, to scavenge ROS as soon as they are generated. However, the excessive production of ROS due to the failure of cellular antioxidant defense systems can result in severe diseases, which has been widely shown to be related in various human pathologies, such as inflammation, diabetes, chronic and neurodegenerative diseases and aging.

### ROS generation in wound healing

Wound healing is the normal biological process in the human body, which is naturally reacted to tissue injuries. In general, an optimal wound healing process is achieved through four precisely and highly programmed phases: hemostasis, inflammation, proliferation and remodeling. These phases should occur in the proper sequence and time frame for a successful wound healing. ROS are involved in all phases of wound healing process. For instance, in hemostasis phase, ROS mediate the vasoconstriction, following platelets exposure to extracellular matrix (ECM)/collagen and thrombin activation, resulting to the thrombus formation to protect the wound and prevent further blood loss. Then, in the second phase, the migration of neutrophils, monocytes toward the wound sites are promoted via ROS signaling to help attack invading pathogen. Also, phagocytosis releases ROS to stunt bacterial growth and provide further signals supporting the wound response. At the third and fourth phase, ROS released from the wound edge stimulate the EC division and migration for blood vessel reformation, as well as promote the division and migration of fibroblast and keratinocyte for new ECM formation (including collagen synthesis). Depending on the produced ROS concentrations during those phases, the wound healing process can be improved or impaired. At a moderate level of ROS, it stimulate the cell migration and angiogenesis, therefore promoting the normal wound healing. However, if the ROS production overwhelm the activities of cellular antioxidant systems, oxidative stress is induced and subsequently stalls wound healing process. For example, the diabetic chronic wounds are caused by the excessive ROS accumulation resulted from high levels of advanced glycation in the blood under persistent hyperglycinemia [29]. The excessive ROS accumulation in the wound can inhibit the function of macrophages and angiogenesis, thus hindering wound tissue regeneration and blood vessel reconstruction [30]. Moreover, the excessive ROS and RNS can directly or indirectly modify and degrade ECM proteins, leading to the impaired fibroblast and keratinocyte functions.

### ROS generation in cardiovascular diseases

Cardiovascular diseases, such as myocardial infarction (MI), rheumatic heart disease, atherosclerosis, thrombosis, pulmonary embolism, stenosis and ischemic heart failure, are the leading cause of mortality, resulting in 31% of deaths worldwide. Although ROS signaling has be proven to play an important role in controlling the normal heart function, the oxidative stress induced by excessive ROS is related to many cardiac pathologies [31]. For instance, in ischemia–reperfusion injury, the mitochondria dysfunction leads to the unregulated ROS production, which causes the apoptosis of ECs, vascular smooth muscle cells (VSMCs) and cardiomyocytes (CMs). The oxidative stress not only causes the damage to the vascular cells but also affects the blood vessels and surrounding tissue, consequently, leads to the similar damages to other tissues and organs. In heart failure, the ROS production is attributed to the chronic neurohormonal activation, upregulation of angiotensin II and the increased myocardial stresses associated with hypoxia. Among these, angiotensin II upregulates the ROS production through NOX2, which causes the CM hypertrophy, and profibrotic and proinflammatory changes in the cardiovascular system. In hypertension, the increased activity of the three subtypes of NOX (NOX1, NOX2 and NOX4) in blood vessels is related to oxidative stress, leading to the dysfunction ECs and VSMCs and further causing the vascular damage [32]. In atherosclerosis, the excessive ROS has also been reported to trigger the lipid peroxidation, promote thrombus formation, as well as inducing the pulmonary vascular lesions and inflammation.

### ROS generation in inflammatory diseases

RONS play important roles in immune system associated with inflammation. As described above, ROS produced by phagocytic cells, such as neutrophils, monocytes and macrophages, or dendritic cells, is essential to kill of pathogen. However, it becomes cytotoxic while overproduction. The oxidative stress induced by excessive ROS causes cellular damage on DNA, protein and lipids, and ultimately leading to the destruction of normal tissue and chronic inflammation. This chronic inflammation is associated with many serious diseases, including atherosclerosis, diabetes, asthma, arthritis, cancer and even obesity. For example, the excessive mitochondrial ROS production progresses the development of atherosclerosis. Similarly, although NO mediates the cardio protection after ischemia/reperfusion, convincing evidence indicates that excessive ROS production can mediate post ischemic injury, which is supported by the increase levels of ROS and lipid peroxidation products in post-ischemic tissues [33]. Moreover, the loss of homeostasis induced by imbalance between the production and scavenging of ROS is responsible for increased cancer incidence. Since oxidative stress has been shown to induce the damage to DNA, lipid and proteins, many cancer types are potentially caused by chronic inflammation and ROS-related mutagenesis. Interestingly, ROS can be used as a double-edged sword for cancer treatment. Many researchers have designed ROS releasing biomaterials, combined with other therapies, such as starving therapy, photothermal therapy and chemotherapy, for a synergistic antitumor effect [34, 35]. In another way, compared to normal cells, cancer cells generate higher level of ROS due to higher metabolic activity and more rapid proliferation of transformed cells. Therefore, ROS-responsive injectable hydrogels become a potential candidate for smart drug delivery systems.

### ROS generation in neurodegenerative diseases

Neuron cells are particularly vulnerable to oxidative stress induced by ROS overproduction, due to their weakened antioxidant defense system and long lifespan or post-mitotic nature. Therefore, ROS overproduction has been shown to associate with many neurodegenerative diseases and several neurological conditions, including Alzheimer’s disease, Parkinson’s disease, amyotrophic lateral sclerosis, neurodegeneration with brain iron accumulation and Huntington’s disease. Mitochondrial dysfunction has been indicated to increase ROS generation, which further promote c-Jun-N-Terminal Kinase (JNK) and Sterol Regulatory Element Binding Protein activity in neurons, resulting to the neurodegeneration through the accumulation of lipid droplets [36]. In addition, different neuronal groups have different intrinsic levels of oxidative stress, therefore more vulnerable to additional disease-related oxidative stress [37]. However, it is also noted that ROS plays an important role in metabolism and energy perfusion of neuron cells, which is essential for their development.

## Injectable RONS-scavenging hydrogels

As mentioned above, oxidative stress induced by excessive ROS levels directly causes damage to cellular components, such as lipids, proteins and DNA, which is well known to be closely related to the development and deterioration of various diseases, aging and cancer [38]. Therefore, the administration of exogenous ROS scavengers to suppress oxidative stress injuries is the most plausible method to treat such physiological disorders. However, the systemic administration of conventional ROS drugs may cause the adverse effects, such as renal toxicity and rapid clearance by the kidney [39]. So far, it is imperative to develop drug delivery systems that can not only prevent side effects but also improve the therapeutic effects of ROS scavengers at the target tissues. Recently, injectable hydrogels have been extensively developed as promising ROS-scavenging platforms by introducing ROS-scavenging molecules and nanomaterials into the gel matrix. ROS-scavenging hydrogels can be obtained by incorporating various ROS-scavenging molecules into the hydrogel matrix. In this review, we focus on two types of injectable hydrogels containing with: (i) inorganic nanoparticles mimicking antioxidant enzymes and (ii) organic antioxidant moieties.

### Injectable hydrogels incorporated with inorganic nanoparticles

Nanomaterials that can mimic the natural antioxidant enzyme (termed as ‘nanozymes’) is one of promising strategy for suppression the development of ROS-related diseases [40–43]. Compared to native enzymes, including CAT, SOD and GPx, such nanozymes have high and broad-spectrum ROS-scavenging capacity, and high stability in the harsh disease environment [44]. Until now, various ROS-scavenging nanozymes have been developed, such as ceria (CeO_2_) nanoparticles, iron oxide (Fe_2_O_3_) and manganese oxide (MnO_2_, Mn_2_O_3_, Mn_3_O_4_ and MnO_2_) nanoparticles and carbon nanomaterials were incorporated into the hydrogel matrix, resulting in antioxidant hydrogels [45, 46].

**Figure 1.**
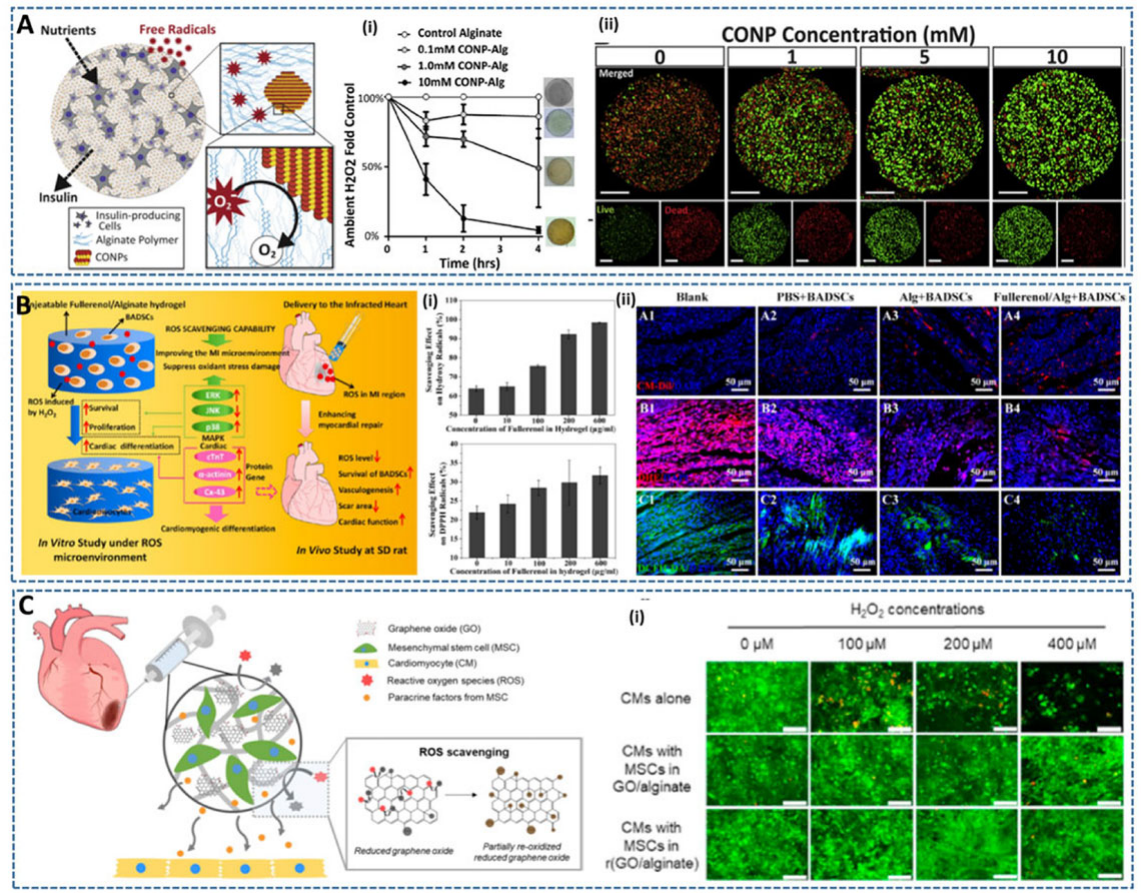
Injectable ROS scavenging alginate-based hydrogels for cell therapy. (**A**) CONP-alginate composite hydrogel: (**i**) H_2_O_2_ scavenging activities of CONP-alginate composite hydrogels loaded with different CONPs concentrations, and (**ii**) cytoprotective effect of CONP-alginate hydrogels as the function of CONP concentration from oxidative stress. Reproduced with permission from Ref. [47] Copyright 2015 Elsevier Ltd. (**B**) Fullerenol/alginate hydrogels: (**i**) ROS scavenging properties of fullerenol/alginate hydrogel against hydroxyl radical and DPPH radicals as the function of fullerenol concentration, and (**ii**) representative images of implanted BADSCs labeled with CM-Dil (red) in MI zone, DHE images (red) and DCFH-DA images (green) of heart section after 24 h post-injection. Reproduced with permission from Ref. [49] Copyright 2017 American Chemical Society. (**C**) Antioxidant rGO/alginate hydrogels: (**i**) the viability of free CMs and encapsulated MSCs, and proliferation of co-cultured CMs in hydrogels at different H_2_O_2_ concentrations. Reproduced with permission from Ref. [50] Copyright 2019 Elsevier Ltd.

Cerium oxide nanoparticles (CONPs) have been widely used as ROS scavenging agents because of their ability to cycle between their cerium (III) and cerium (IV) oxidation states, which can exhibit catalytic activities of SOD and CAT enzymes, respectively, with self-renewing properties [47]. However, the cytotoxicity of CONPs induced by cellular internalization and accumulation of nanoparticles has also been observed. Therefore, Weaver *et al.* [47] encapsulated CONPs in an injectable alginate hydrogel to mitigate the cytotoxicity as well as retain their catalytic potential at the target site (Fig. 1A). In this study, CONPs showed the protective effects for beta cells (MIN6 cells) against the oxidative damage of superoxide radicals, depending on the CONPs concentration (0.1–10 mM) [47]. Although the increase of CONPs concentration increased the protective effect, it also showed a decreasing trend in the metabolic activity of beta cells. Fortunately, after encapsulation in the alginate hydrogel, the reactivity of CONPs was highly retained for several weeks and the cytotoxicity effect of encapsulated CONPs was decreased by reducing the cellular internalization of nanoparticles. Interestingly, a significant cytoprotective effect of CONP-alginate gels on embedded cells from external oxidative stress in a dose-dependent manner of CONPs concentration was observed, without statistical variation from untreated controls. The notable advantage of this approach is the capacity to self-renew of CONPs, which imparts the nanocomposite hydrogels long-term and continuous protective effects to prevent cell death post-transplantation. However, the biocompatibility and effectiveness of this system need to be fully validated *in vivo*.

Recently, CONPs and antimicrobial peptide were loaded into a sprayable gelatin-based hydrogel to combine ROS scavenging and antibacterial properties for wound management [48]. Herein, dopamine was conjugated to the backbone of photocrosslinkable gelatin methacryloyl to produce a strong adhesive hydrogel wound dressing. The synergistic effects of this hydrogel, including sprayability, adhesiveness, and most importantly, antimicrobial and ROS-scavenging activities, were proven to not only increase the wound healing speed but also promote re-modeling of healed skin under infectious and inflammatory conditions. Therefore, this multifunctional hydrogel with a simple production process may possess a significantly high potential to address the therapeutic and economic burdens associated with chronic wound treatment and management.

In another study, Wang *et al*. [49] encapsulated fullerenol nanoparticles into alginate hydrogels to create an injectable cell delivery scaffold with antioxidant activity (Fig. 1B). Compared to other carbon nanomaterials (e.g. graphene and carbon nanotube), fullerenol nanoparticles have excellent water solubility with much lower cytotoxicity, which is beneficial to prepare homogeneous nanocomposite hydrogels. Their results showed that the fellurenol/alginate hydrogel not only protected brown adipose-derived stem cells (BADSCs) seeded in hydrogels from oxidative stress damage but also improved the cardiomyogenic differentiation of BADSCs even under the ROS microenvironment. The *in vivo* therapeutic effect of the fullerenol/alginate hydrogel was evaluated by implanting hydrogel-formulated BADSCs into the MI area in rats. In particular, the molecular mechanism of the cytoprotective effect of the hydrogels was elucidated by investigating the expression of extracellular signal-regulated kinase (ERK) pathways, including ERK, JNK and p38, which are related to stem cell survival, proliferation, apoptosis and cardio myogenesis.

Similarly, Choe *et al.* [50] developed an antioxidant graphene oxide (GO)/alginate nanocomposite microgel to encapsulate mesenchymal stem cells (MSCs), which has the potential to protect MSCs from the high oxidative stress in MI diseases (Fig. 1C). Notably, the GO embedded in hydrogels was further chemically reduced GO (rGO) instead of using pre-reduced GO, which can avoid the aggregation of insoluble rGO while improving the antioxidant capability of GO with minimal potential to the cells. The results showed that the incorporation of rGO greatly increased the scavenging activities of alginate and GO/alginate hydrogels in multiple assays (ABTS, DPPH, H_2_O_2_ and superoxide assays), depending on the reduction time and rGO content. Interestingly, the MSCs encapsulated in (r)GO/alginate hydrogels showed increased viability and protected CMs from high oxidative stress and supported their cardiac differentiation by the positive paracrine signals from MSCs. The *in vivo* experiments also confirmed the therapeutic effect of the MSC encapsulated (r)GO/alginate system in improving the remodeling after 2 weeks of MI, compared to infarcted hearts treated with either MSCs or MSC in GO/alginate. However, it is necessary to observe this for longer time periods to thoroughly evaluate the effect of MSC delivery and possible GO or rGO-associated toxicities for the treatment of infarcted heart.

**Figure 2.**
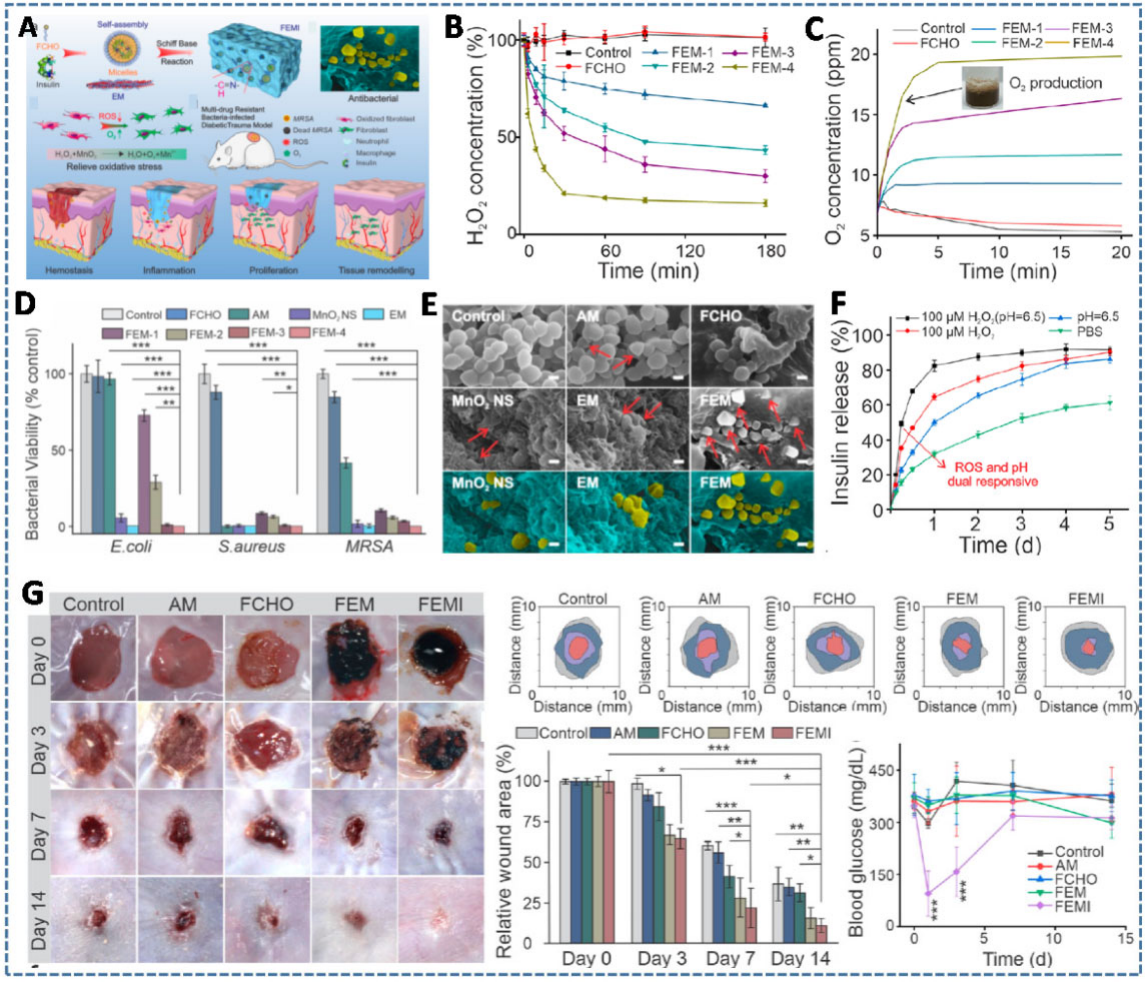
(**A**) Schematic illustration of MnO_2_ and insulin embedded hydrogel (FEMI) for accelerating wound healing process of MRSA-infected diabetic wound. (**B–E**) The profiles of H_2_O_2_ scavenging, O_2_ generation and antibacterial activities of hydrogels as the function of MnO_2_ concentration. (**F**) Dual pH and redox responsive insulin release characteristics from hydrogel. (**G**) The synergistic effect of FEMI hydrogel on wound healing process compared to other control groups (Phosphate buffered saline (PBS), antibiotic ampicillin, FCHO, FEM), demonstrated by traces and rates of wound-bed closure and the changes in blood glucose level. Reproduced with permission from Ref. [51] Copyright 2020 American Chemical Society.

Manganese dioxide (MnO_2_), a novel nanoenzyme that catalyzes the decomposition of H_2_O_2_ into O_2_, has been proven to be a promising candidate for assuaging both oxidative and hypoxic microenvironments. Taking these advantages, Wang *et al.* [51] embedded insulin and MnO_2_ nanosheets into an ε-polylysine (EPL)-based injectable hydrogel, to develop a multifunctional hydrogel with antibacterial, hyperglycemic manipulation, ROS scavenging and oxygen production activities to facilitate the diabetic wound healing (Fig. 2). The hydrogel was formed via a Schiff-based reaction between the amino groups of EPL-coated MnO_2_ nanosheets and the aldehyde groups of insulin-encapsulated Pluronic F127 micelles (FEMI hydrogel). Particularly, the use of MnO_2_ nanosheets not only alleviated the oxidative stress and hypoxia in diabetic skin circumstance but also improved the rheological properties and antimicrobial capacity of the resulting hydrogel via ‘nanoknife-like’ effect. The results demonstrated a clear MnO_2_ nanosheet concentration-dependent H_2_O_2_ decomposition, O_2_ production, mechanical properties and antibacterial abilities. In addition, the decomposition of the MnO_2_ nanosheets through H_2_O_2_ facilitated redox reaction also contributed to the degradation of the hydrogels, which accelerated the release of encapsulated insulin to regulate the blood glucose. The synergistic effect of the FEMI hydrogel on bacterial inhibition and wound healing was examined by using a methicillin-resistant *Staphylococcus aureus* (MRSA)-infected diabetic trauma model, which showed the most effective bactericidal action and wound closure rate compared with the other control groups.

### Injectable hydrogels conjugated with organic antioxidant moieties

There are numerous organic compounds that can eliminate ROS via electron transfer or proton donation, such as polyphenols, boronic acid, aniline or sulfur-containing amino acids [17]. Depending on the hydrogel design and the ROS scavenging mechanism, these compounds can be physically incorporated or chemically conjugated in the hydrogel matrix. Compared to the physical incorporation, the covalent bonding of these organic compounds onto the precursor polymers provides sustainable and long-term ROS scavenging activities, as well as reduces the adverse effects due to the burst release of ROS scavengers.

**Figure 3.**
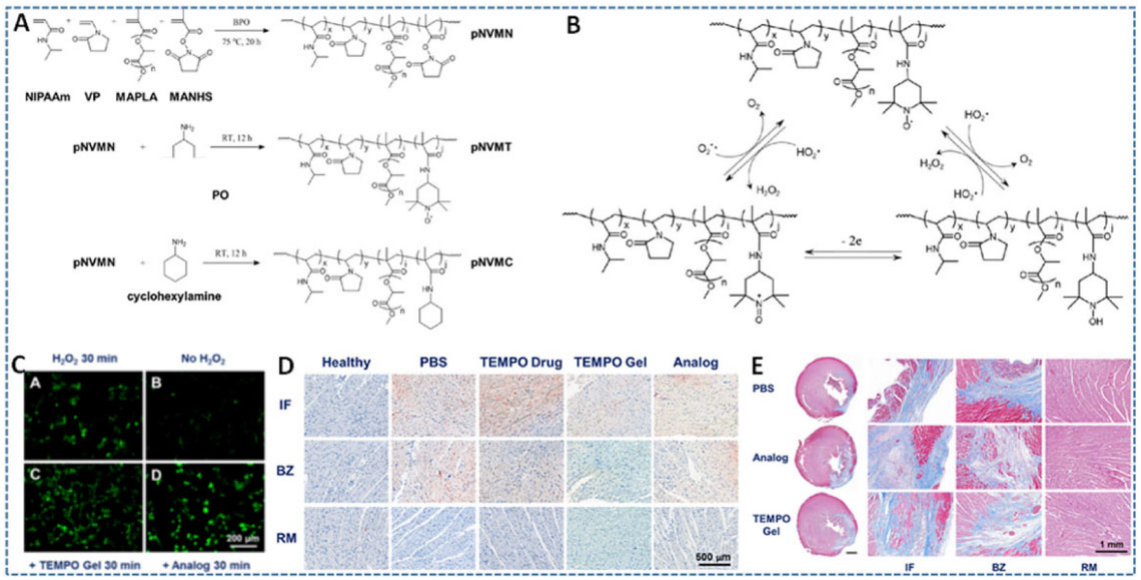
(**A** and **B**) Synthesis images and ROS scavenging mechanism and recyclability of thermally responsive polymer with TEMPO pendant groups. (**C**) The cell protective effect of hydrogels against oxidative stress induced by H_2_O_2_ treatment. (**D**) Cell apoptosis 1 day after infarction/injection/reperfusion in infarction area (IF), border zone (BZ) and remote area (RM) and (**E**) the ventricular wall histology of rat heart after 8 weeks of treatment with PBS, analog and TEMPO gel stained by masson’s trichrome. Analog is hydrogel formed by poly(NIPAAm-co-VP-co-MAPLA-co-MACHA) (pNVMC) polymer as control hydrogel sample. Reproduced with permission from Ref. [39] Copyright 2018 Elsevier Ltd.

Zhu *et al.* [39] conjugated a recyclable ROS scavenging nitroxide radical, TEMPO (4-amino-TEMPO or 4-Amino-2,2,6,6-tetramethylpiperidine-1-oxyl), onto the backbone of a thermally responsive polymer to achieve an injectable hydrogel with highly efficient ROS scavenging activities (TEMPO Gel) (Fig. 3). The covalent bonding of TEMPO to the polymer not only provided sufficient contact between the TEMPO moieties and the tissue but also extended the local retention of TEMPO at the target tissues. The significant ROS scavenging characteristics of this hydrogel was attributed to the recyclability and high efficiency of the nitroxides. TEMPO Gel can scavenge a variety of ROS, including hydroxyl and superoxide radicals, and improve the survival of smooth muscle cells under oxidative stress. *In vivo* rat MI/reperfusion model also indicated that TEMPO Gel can protect the myocardium from oxidative injury and result in long-term improvements in left ventricle geometry, compared to both Analog gel and PBS treatments without ROS scavenger as controls. Interestingly, the nitroxide in TEMPO endowed the hydrogels with paramagnetism, allowing the *in vivo* magnetic resonance imaging of the injected hydrogel. Although this TEMPO Gel showed potential in mitigating complications of ROS-related diseases in soft tissue pathological conditions, it still remained limitations, such as the non-scavenging intracellular ROS ability due to the covalent bonding of TEMPO on the polymer backbone, and the lack in design of experiments, including the effect of TEMPO concentration on the scavenging effects of hydrogel, and the evaluations for secondary of injuries induced by the hydrogel injection procedure and hydrogel diffusion into the soft tissues.

**Figure 4.**
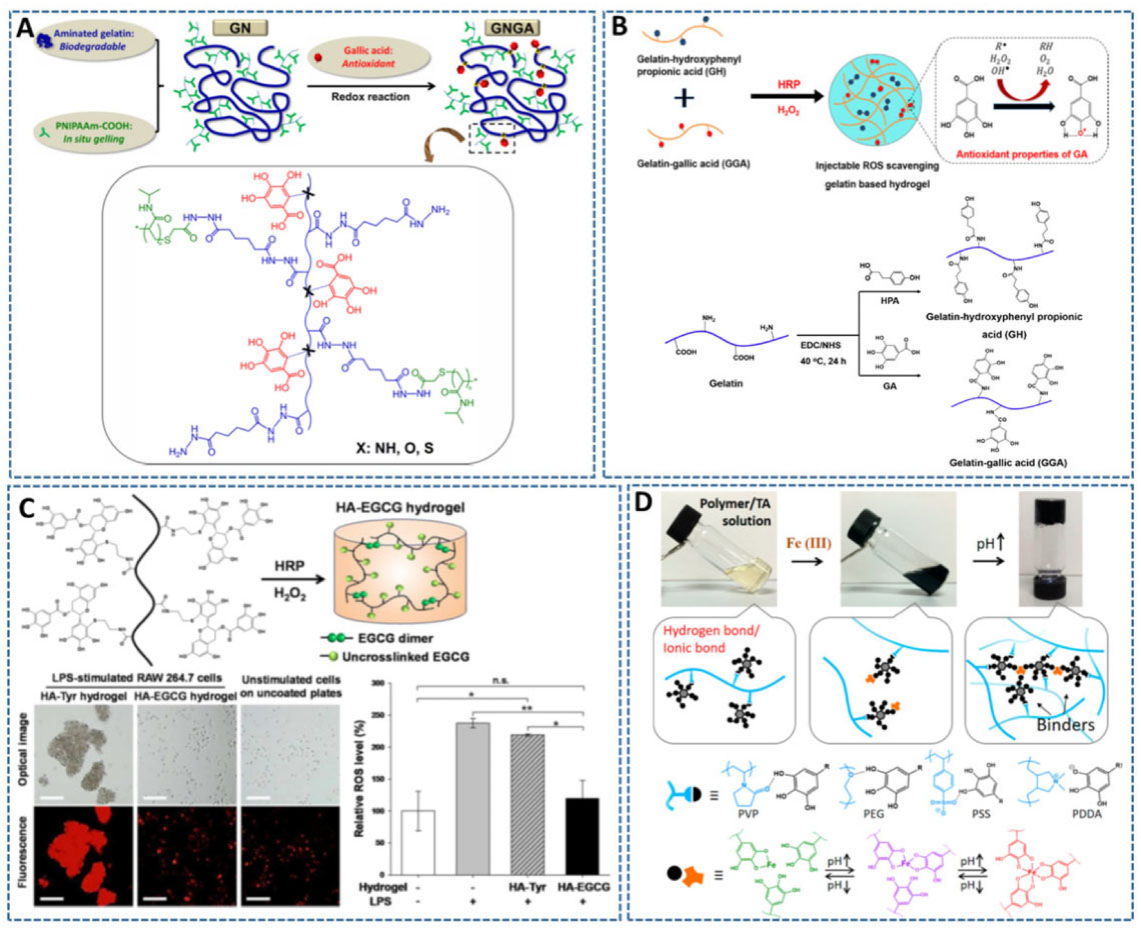
Development of antioxidant injectable hydrogels by using polyphenols. (**A**) The synthesis of multifunctional polymer by grafting GA molecules and thermoresponsive poly (nisopropylacrylamide) (PNIPAAm) to the biodegradable gelatin backbone. Reproduced with permission from Ref. [54] Copyright 2015 American Chemical Society. (**B**) The incorporation of GA-gelatin conjugates into horseradish peroxidase (HRP)/H_2_O_2_-catalyzed *in situ* forming hydrogels endows hydrogels cytoprotective activities against H_2_O_2_-induced ROS microenvironment, which efficiently accelerated the wound healing process in a mouse skin defect model. Reproduced with permission from Ref. [55] Copyright 2019 Elsevier Ltd. (**C**) The coupling between EGCG moieties of HA-EGCG polymers catalyzed by enzymatic crosslinking reaction of HRP and H_2_O_2_, resulting to HA-EGCG hydrogel that can reduce the intracellular ROS generation from macrophage (lipopolysaccharide (LPS)-stimulated RAW 264.7 cells). Reproduced with permission from Ref. [60] Copyright 2017 American Chemical Society. (**D**) Supramolecular hydrogels with rapid self-healing and pH-responsiveness based on interactions between polymer/TA/Fe(III) complexes. Reproduced with permission from Ref. [61] Copyright 2017 American Chemical Society.

Polyphenols, such as gallic acid, tannic acid (TA), epigallolcatechin-3-O-gallate (EGCG) and quercetin, have been found to be strong antioxidants, therefore having attracted much attention to be incorporated into injectable hydrogels to endow hydrogels with antioxidant activities [52, 53]. Recently, the conjugation of gallic acid (GA) onto macromolecules, such as gelatin, has been reported to retain the antioxidant activity of GA and reduce the adverse effects caused by the burst leaching of small GA from the hydrogel matrices [54, 55]. These GA-functionalized gelatin-based hydrogels enhanced the cytoprotective effects of hydrogels against the H_2_O_2_-induced oxidative stress microenvironment. The *in vivo* findings from these studies also showed the advantages of antioxidant hydrogels for the improvement of the total antioxidant status in chronic ocular hypertension and wound progression, thereby enhancing the therapeutic effects over their pure gelatin counterparts (Fig. 4A and B). Beside gelatin, GA also was conjugated onto the chitosan backbone (CGA) and served as both antioxidant and crosslinking moieties for *in situ* formation of antioxidant hydrogels [56]. The antioxidant activities of CGA hydrogels were demonstrated by their scavenging effects against DPPH radicals, hydroxyl radicals and total reducing power. Notably, CGA hydrogels significantly improved the viability and migration of fibroblasts owing to the high toxicity of ROS-induced oxidative stress triggered by hyperglycemia. Although *in vivo* experimental data were not presented, this CGA hydrogel is expected to be a potential material for the treatment of diabetes-related diseases.

Similar to gallic acid, TA and EGCG have gained significant attention for their excellent biological properties, such as antioxidant, free radical scavenging, anti-inflammatory and antibacterial activities [57, 58]. In recent years, TA and EGCG have been widely used as ideal crosslinkers for *in situ* formation of hydrogels, owing to their ability to complex or crosslink macromolecules at multibinding sites through hydrogen/ionic bonding and hydrophobic interactions [59]. In particular, beside antioxidant property, the resulting hydrogels exhibit multiple functionalities, including adhesiveness, self-healing, antibacterial, anti-inflammatory and degradation resistance. For example, Kurisawa *et al*. [60] developed a long-lasting injectable hydrogel by conjugating EGCG onto HA polymer (Fig. 4C). In contrast to unmodified HA hydrogels, *in situ* forming HA-EGCG hydrogels possessed free radical scavenging activities and high resistance to hyaluronidase-mediated degradation with minimal inflammation when subcutaneously injected into mice. These properties are attributed to the ability of EGCG moieties to quench free radicals and bind to enzymes via hydrogen bonding, π–π stacking and hydrophobic interactions, thereby attenuating foreign-body reactions at the hydrogel-tissue interface by scavenging macrophage-derived ROS, and inhibiting hyaluronidase-mediated degradation [60]. Likewise, Fan *et al.* [61] exploited the versatile bonding abilities of TA to design a series of supramolecular hydrogels formed by commercially available water-soluble polymers, including polyvinylpyrrolidone, poly-(ethylene glycol) (PEG), poly(sodium 4-styrenesulfonate) and poly(dimethyldiallylammonium chloride) (Fig. 4D). In these hydrogels, TA interacts with polymers via H-bonds or ionic bonds and connects to each other via coordinate bonds in the presence of Fe (III) ions. By tuning the weight ratios of polymer/TA and TA/Fe^3+^, the interactions between these three components can be elegantly balanced for the construction of hydrogels with wide adjustability in mechanical strength. Although the interaction between TA and polymer/Fe^3+^ reduced the free scavenging effects of hydrogels compared to free TA molecules, these TA-polymer gels still possess remarkable free-radical-scavenging ability against DPPH and ABTS radicals [61]. The notable advantages of these TA based hydrogels are easy and green processing, low cost, and large-scale preparation. However, further *in vitro/in vivo* cytotoxicity and cytoprotection of hydrogels need to be performed to evaluate the effect of the antioxidant properties of hydrogels for potential applications.

**Figure 5.**
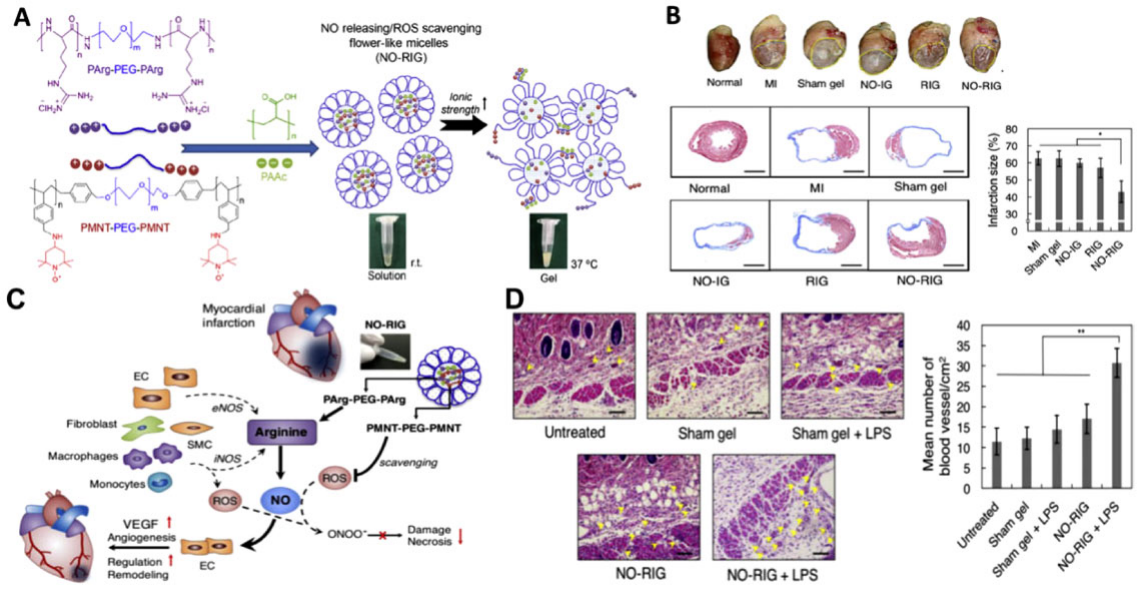
(**A**) The schematic presentation for the formation of NO releasing/ROS scavenging injectable hydrogel (NO-RIG). (**B**) The proposed mechanism for sustained release of NO and the ROS scavenging ability of NO-RIG, which significantly prevents the progressions of MI and improves cardiac functions. (**C**) The therapeutic effect of NO-RIG in myocardial infraction (MI) mice demonstrated by the gross observation of the heart and the infarction size by Masson Trichrome staining, compared with control gels such as sham (no NO generation and no ROS scavenging), NO-IG (NO generation but not ROS scavenging) and RIG (no NO generation but ROS scavenging). (**D**) H&E staining of the abdominal skin at the gel injected sites and number of blood vessels near the gel injected sites. Scale bars = 100 µm. Reproduced with permission from Ref. [62] Copyright 2018 Elsevier Ltd.

Interestingly, Vong *et al.* [62] developed a smart redox-injectable hydrogel that can simultaneously release NO and scavenge the overproduced ROS in the MI environment. This study explored the important role of NO in protecting and regulating cardiovascular functions, as well as preventing the reaction between NO and evaluated ROS during cardiovascular disease, which can further induce tissue injury. For this purpose, they combined two triblock copolymers, poly(L-arginine)-poly(ethylene glycol)-poly(L-arginine) (PArg-PEG-PArg) as the NO-releasing polymer and poly[4-(2,2,6,6-tetramethylpiperidine-N-oxyl)aminomethylstyrene]-b-poly(ethylene glycol)-b-poly[4-(2,2,6,6-tetramethylpiperidine-N-oxyl)aminomethylstyrene] (PMNT-PEG-PMNT) as the ROS-scavenging polymer, coupled with poly(acrylic acid) (PAAc). These polyion complexes (PICs) formed so-called flower-type micelles because of the loop structure of the PEG chain as a shell and were converted to hydrogel as the temperature increased because of the partial destabilization of PIC micelles (Fig. 5). Compared to control injectable NO-releasing or ROS-scavenging hydrogels, the NO-releasing redox injectable hydrogel (NO-RIG) remarkably decreased the infarction size and improved the heart function after MI, attributed to the regulation of NO sustained release and redox equilibrium at the locally injected sites. This NO-RIG hydrogel has great potential for the treatment of cardiovascular diseases by encapsulating versatile drugs (e.g. biomolecules, drugs, cells) for synergistic therapeutic efficiency.

## Injectable RONS-responsive hydrogels

Biomolecule-responsive hydrogels that can respond to the target biomolecules, such as DNA, peptides, enzymes, proteins and small molecules, have attracted substantial attention as smart biomaterials for drug delivery systems and molecular regulators for homeostasis in living organisms [63–66]. Since RONS are known to be involved in the pathogenesis of various diseases, RONS-responsive hydrogels hold great potential as matrices to trigger the release of therapeutic drugs and eliminate excess RONS levels for relieving the cellular damage [26, 67]. Depending on the materials or the design of hydrogels, the therapeutic drugs can be released from the hydrogel matrices through either ROS-induced solubility change or ROS-induced cleavage. There are many kinds of ROS-sensitive materials and linkers are used for drug delivery systems, such as thioether, thioketal, boronic ester, polyproline, selenium/tellurium, etc. The chemical structures and oxidation mechanisms of these ROS-sensitive materials are briefly summarized in Table 1, which have been thoroughly discussed in previous reviews [26, 68, 69]. The following section introduces recent RONS-responsive hydrogels and discusses their design principles and *in vitro/in vivo* evaluations, by utilizing RONS-responsive materials and linkers for different therapeutic purposes.

**Table 1.** The chemical structures and oxidative mechanisms of ROS-responsive materials for drug delivery systems

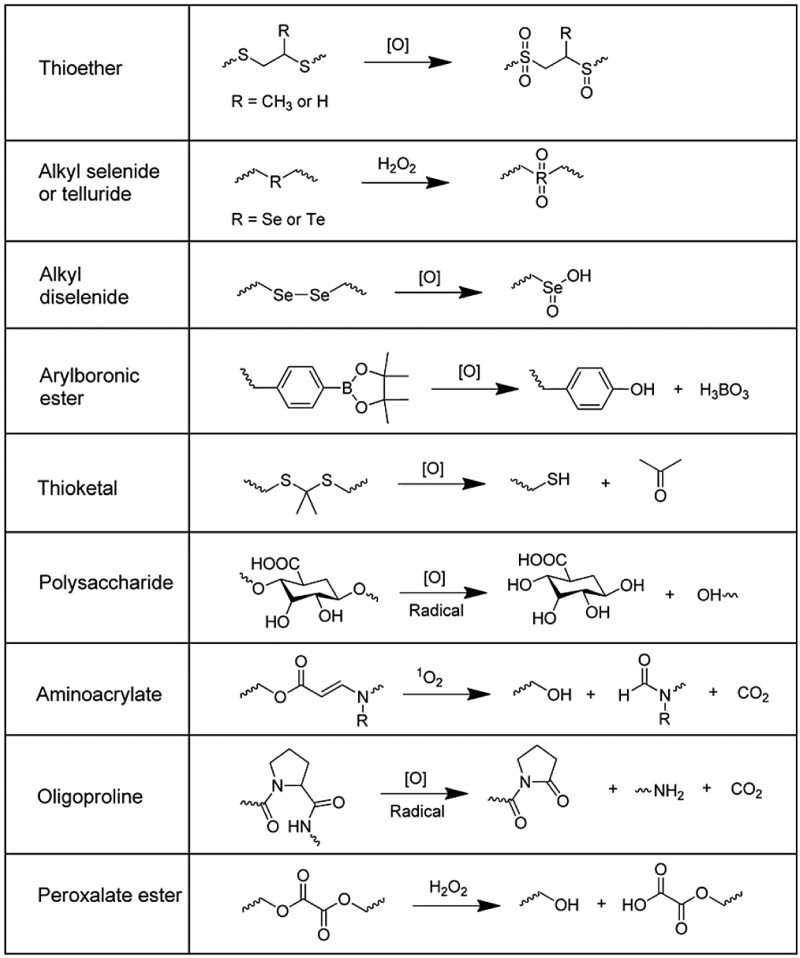

Reproduced with permission from Ref. [68] Copyright 2016 Wiley-VCH.

### Injectable ROS-responsive hydrogels

**Figure 6.**
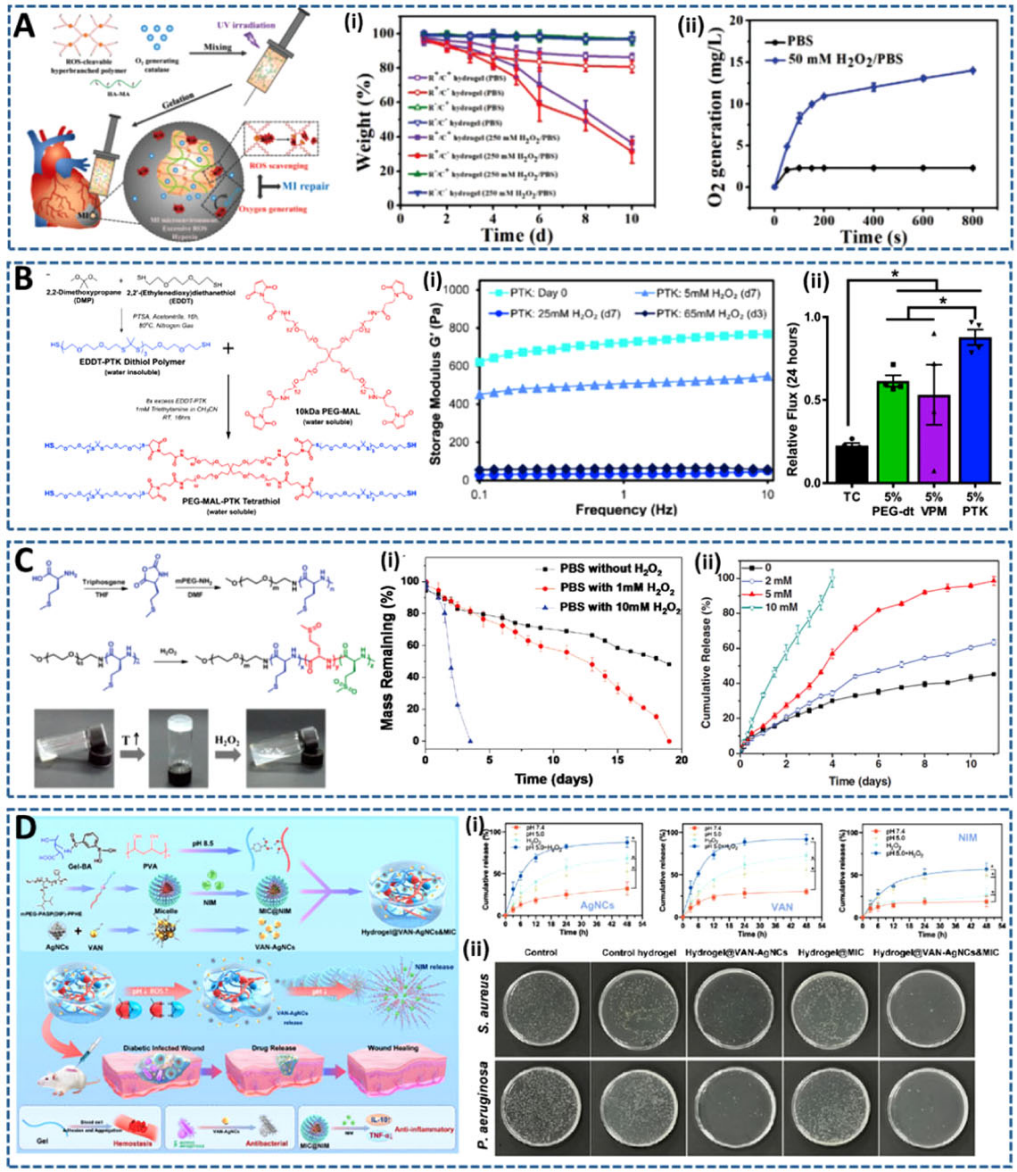
Injectable ROS-responsive hydrogels for triggering drug release. (**A**) ROS-cleavable HA based hydrogels for O_2_ generation: (**i**) the degradation behaviors of ROS responsive hydrogels with (R^+^/C^+^) or without CAT (R^+^/C^−^) and ROS-nonresponsive hydrogels with/without CAT (R^−^/C^+^ and R^−^/C^−^) in the presence/absence of H_2_O_2_, and (**ii**) O_2_ generation of R^+^/C^+^ hydrogels in PBS and H_2_O_2_/PBS solutions. Reproduced with permission from Ref. [70] Copyright 2020 Wiley-VCH. (**B**) PTK crosslinked hydrogel for H_2_O_2_-mediated and dose dependent degradation: (**i**) the mechanical integrity loss of hydrogels in response to H_2_O_2_, and (**ii**) ROS cytotoxicity protection of PTK gel compared to 2D tissue culture (TC) and PEG-dt and peptide crosslinked gels, determined by total viable cell number 24 after exposure to H_2_O_2_. Reproduced with permission from Ref. [71] Copyright 2020 Elsevier Ltd. (**C**) Thermo- and ROS-responsive hydrogel composed of mPEG-PMet copolymer: (**i**) the H_2_O_2_-dependent degradation behavior of hydrogels, and (**ii**) rhodamine 6G released from hydrogels, which is triggered by H_2_O_2_ levels. Reproduced with permission from Ref. [72] Copyright 2016 Wiley-VCH. (**D**) Multifunctional hydrogel encapsulating NIM-loaded micelle and VAN-AgNCs (hydrogel@VAN-AgNC&MIC): (**i**) the pH and ROS-responsive behaviors of hydrogel for subsequent and sustained release of AgNCs, VAN and NIM, and (**ii**) antibacterial effect of hydrogels against both *S. aureus* and *Pseudomonas aeruginosa* (*P. aeruginosa*). Reproduced with permission from Ref. [73] Copyright 2021 American Chemical Society.

Ding *et al.* [70] developed a novel injectable hydrogel formed using synthetic ROS-cleavable hyperbranched polymers to release CAT for O_2_ generation. This idea is initiated from the oxidative stress and hypoxia, two main characteristics, in the tissue microenvironment after MI. Therefore, they chose thioketal linkages, which are sensitive to $O_{2}^{-}$ and H_2_O_2_ produced after MI, to synthesize ROS-cleavable and consumable hyperbranched polymers (HBPAK). The hydrogels were rapidly formed within 3 s by copolymerization with methacrylate hyaluronic acid (HA-MA) under UV irradiation. During hydrogel formation, CAT is entrapped to convert H_2_O_2_ to O_2_ (Fig. 6A). Using this approach, the hydrogel was conveniently injected into the infarcted rat heart, which effectively released CAT through ROS-triggered hydrogel degradation. Therefore, the hydrogel plays dual functions of scavenging excessive ROS and releasing O_2_ to simultaneously alleviate the inflammation and hypoxia in MI microenvironment. These functions were confirmed *in vivo* by the decrease in ROS-related free radical levels and cell apoptosis in the infarcted heart treated with the hydrogel. Importantly, this hydrogel significantly promoted angiogenesis, reduced the infarcted area and improved cardiac function, compared to ROS or CAT-absent hydrogels.

Martin *et al.* [71] also used thioketal chemistry to design an ROS-degradable PEG hydrogel for stem cell delivery. This study aimed to develop an alternative enzymatically degradable PEG hydrogel using matrix metalloproteinase (MMP)-cleavable sequence linkers. Compared to MMP-cleavable linkers, the synthesis of ROS-degradable polymers is low cost and is specifically degraded by elevated ROS produced from many normal biological signaling cascades. To this end, they synthesized a water-soluble, thiolated poly(thioketal) (PTK) that efficiently reacted with maleimide-functionalized PEG (PEG-MAL) via a Michael-addition reaction for the *in situ* formation of hydrogel (Fig. 6B). As expected, the PTK-crosslinked hydrogel showed ROS-mediated and dose-dependent degradation *in vitro*, as demonstrated by the H_2_O_2_ degradation-mediated mechanical integrity loss. Notably, the viability of mouse MSCs encapsulated in PTK-based hydrogels was maintained significantly than those in gels crosslinked with either commercially available PEG dithiol (PEG-dt) or enzymatically degradable peptide (VPM), both *in vitro* and *in vivo*. This cellular survival enhancement is supported by the inherently antioxidative behavior of PTK polymers, which have been shown to protect encapsulated MSCs from toxic levels of exogenous ROS. The results from this study open a new paradigm of cell-degradable hydrogels for clinically relevant applications, particularly for cell delivery therapies.

In another study, Xu *et al*. [72] presented an ROS-responsive hydrogel composed of methoxy poly(ethylene glycol)-poly (L-methionine) diblock copolymer (mPEG-PMet). Herein, the incorporation of antioxidative L-methionine residues into the amphiphilic PEG-based block copolymer system not only induced thermoresponsive hydrogel formation but also endowed the resulting hydrogel with ROS-responsibility due to the hydrophobic-to hydrophilic transition after oxidation by ROS (Fig. 6C). To demonstrate the ROS sensitivity of hydrogels, they investigated the H_2_O_2_-responsive degradation process of hydrogels and showed an accelerated release profile of Rhodamine 6G as a model drug from hydrogels, in response to H_2_O_2_. In addition, this mPEG-PMet hydrogel had cytoprotective ability by reducing cell apoptosis under H_2_O_2_-induced oxidative stress microenvironment. Moreover, the hydrogel could also be responsive to physiologically relevant ROS, demonstrated by the accelerated erosion of hydrogel by H_2_O_2_ produced by activated macrophages. This ROS-responsive hydrogel holds great potential for the treatment of ROS-related diseases, where it can eliminate excess ROS to relieve cellular damage and trigger the release of therapeutic drugs under the pathophysiologic oxidative microenvironments.

Very recently, Wang *et al.* [73–75] designed several novel hydrogel systems with innovative characteristics, including injectability, self-healing and, most importantly, sequential and on-demand delivery of antibacterial and anti-inflammatory drugs for enhanced wound healing, by utilizing the low pH and accumulated ROS stimuli that occur naturally in chronically infected wounds. To this end, they fabricated ROS sensitive hydrogels based on the dynamic phenylboronic acid-diol ester bonds between the phenylboronic acid-modified gel (Gel-BA) and poly(vinyl alcohol) (PVA) solution (containing rich o-diols). Thereafter, multidrug-resistant vancomycin conjugated ultrasmall Ag nanoclustuters, VAN-AgNCs, with enhanced antimicrobial efficiencies were encapsulated in the hydrogel, which was promptly released under the circumtances of inflammatory acid and high ROS. pH-responsive miccelle loaded nimesulide, NIM, an anti-inflammatory drug, was also co-encapsulated into the hydrogel, to guarantee the sustained release of NIM after the release of antibacterial VAN-AgNCs. By this way, they created a smart, dual pH, and ROS-responsive hydrogel for sequential release of antibacterial and anti-inflammatory drugs, which effectively kill bacteria and suppress the inflammation to improve wound healing process (Fig. 6D) [73]. The *in vitro* biocompatibility, hemostasis and antibacterial and anti-inflammatory properties of hydrogels were thoroughly investigated. The *in vivo* effects of these properties were confirmed using a chronically infected diabetic wound model, which demonstrated the great ability of the inflammation-responsive hydrogel to accelerate wound closure rate, inhibit inflammation, stimulate new vessel formation and enhance collagen deposition. This smart hydrogel has potential applications as an advanced drug delivery depot for the treatment of various inflammation-associated diseases, where ROS are overexpressed.

Another ROS-scavenging PVA based hydrogel loaded with therapeutic agents for difficult-to heal diabetic wounds was also reported by Liu *et al*. [76]. The hydrogel was rapidly formed by simply mixing PVA with N^1^-(4-boronobenzyl)-N^3^-(4-boronophenyl)-N^1^, N^1^, N^3^, N^3^-tetramethylpropane-1, 3-diaminium (TPA), an ROS-responsive linker. Both mupirocin, an antibiotic that prevents bacterial infection, and granulocyte-macrophage colony-stimulating factor (GM-CSF), a growth factor that promotes tissue regeneration, were co-encapsulated into the hydrogel. The *in vitro* and *in vivo* results showed the advantage of hydrogels in scavenging the excessive ROS generated from the infected diabetic wound, which promoted wound healing by down-regulating pro-inflammatory cytokines, up-regulating M2 phenotype macrophages and promoting angiogenesis and collagen production. Importantly, along with the ROS scavenging process, the hydrogel was gradually degraded to trigger the release of mupirocin and GM-CSF, which enhanced wound healing efficacy by inhibiting bacterial infection and accelerating wound repair, respectively.

### Injectable RNS-responsive hydrogels

**Figure 7.**
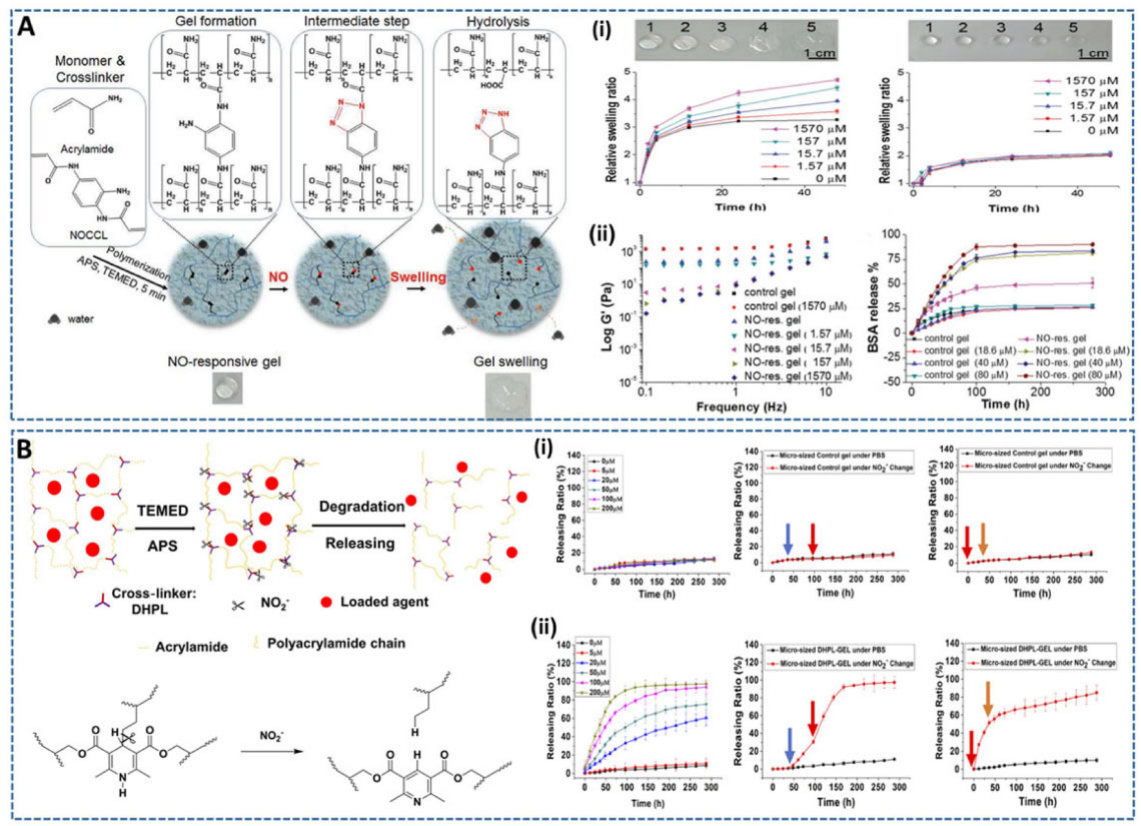
Injectable RNS-responsive hydrogels for triggering drug release. (**A**) NO-responsive hydrogel crosslinked by NOCCL: (**i**) the swelling behaviors of NO-responsive gel (left) and control gel (right) as the function of NO concentration, and (**ii**) the rheological properties of hydrogel and BSA release profiles from hydrogels by varying the NO concentration. Reproduced with permission from Ref. [77] Copyright 2017 Wiley-VCH. (**B**) NO_2_-responsive hydrogels crosslinked by a three-arm dihydropyridine crosslinker (DHPL-GEL). The cumulative releasing ratio and real-time response of liposome from (**i**) control hydrogel and (**ii**) micro-sized DHPL-GEL at various ${\text{NO}}_{2}^{-}$ concentrations. Blue, red, yellow arrows: adjust ${\text{NO}}_{2}^{-}$ concentrations to 50 μM, 200 μM and 20 μM, respectively. Reproduced with permission from Ref. [79] Copyright 2020 American Chemical Society.

The first NO-responsive hydrogel was reported by Kim *et al*. [77], in which a NO-cleavable crosslinker N, N-(2-amino-1,4-phenylene) diacrylamide (NOCCL) was incorporated into an acrylamide-based hydrogel (Fig. 7A). The hydrogel was expected to react with high NO concentrations at the sites of disease in the body, resulting in the triggered release of therapeutic drugs loaded into the hydrogel. The results showed that the hydrogel had rapid responsiveness and high sensitivity and selectivity toward gaseous NO, which led to high swelling and significant change in the morphology of the gel after the injection of NO gas. Furthermore, this NO-responsive gel can be converted to an enzyme-responsive gel by loading an enzyme-responsive NO donor, β-galactosidase-triggering NO donor (β-gal-pyNO). Using this approach, the hydrogel was capable of generating NO when triggered by specific stimuli, namely β-galactosidase enzyme, and the swelling behavior of the gel was subsequently monitored. Importantly, the hydrogel was responsive to the NO secreted from activated macrophage (RAW 264.7), implying a wide range of biomedical applications of the gel. This NO-responsive hydrogel is therefore expected to be useful in many applications, such as drug-delivery vehicles, inflammation modulators and tissue scaffolds for tissue regenerative medicines. Continued with this study, they recently prepared an NO-scavenging nanosized hydrogel (NO-Scv gel) by incorporating NOCCL during acrylamide polymerization for treating rheumatoid arthritis (RA) [78]. This study was motivated by the excess production of endogenous NO from activated macrophages, which causes severe chronic inflammatory disorders in RA. As expected, the NO-Scv gel significantly reduced the inflammatory levels of activated macrophages through its remarkable NO-scavenging capability. Moreover, NO-Scv exhibited excellent therapeutic effects compared to the commercial drug, dexamethasone after intra-articular administration into the paw of RA model mice. Taken together, these results indicate that this nanogel, with easy injectability and high NO-scavenging ability, is likely to be a potential material for treating various inflammation-associated diseases.

A novel nitrite (${\text{NO}}_{2}^{-}$)-responsive hydrogel for smart drug release, depending on the severity of NO-related diseases, was introduced by Liang *et al*. [79]. Compared to other NO-triggered drug-releasing platforms, this (${\text{NO}}_{2}^{-}$)-responsive hydrogel is predicted to be more sensitive because of the higher concentration of ${\text{NO}}_{2}^{-}$ than NO *in vivo* (10–100 times). In addition, the response of the micro-sized hydrogel was also more sensitive than that of the macro-sized hydrogel because of the larger specific area of the micro-sized gel. Therefore, they designed a micro-sized, NO_2_-responsive hydrogel for the construction of an on-demand drug delivery system to advance the treatment of NO-related diseases (Fig. 7B). Accordingly, a three-arm dihydropyridine crosslinker (DHPL), which shows good selectivity and a high yield in NO_2_-induced cleavage, was synthesized to crosslink acrylamide for hydrogelation (DHPL-GEL). *In vitro* and *in vivo* release tests demonstrated the high sensitivity of DHPL-GEL to different concentrations of ${\text{NO}}_{2}^{-}$, which led to the accelerated release rate of loaded liposomes. To test the feasibility of NO_2_-responsive hydrogels for the treatment of NO-related diseases, methotrexate liposome (MTX Lipo), an antirheumatic drug, was encapsulated into the micro-sized DHPL-GEL (MTX Lipo. &DHPL-GEL) and injected into the hind paws of an arthritis rat model. The results showed the superior therapeutic efficacy of MTX Lipo. &DHPL-GEL than the other control groups, demonstrated by the complete inhibition of disease progression and better bone recovery. These positive results suggest the potential of micro-sized NO_2_^-^ responsive hydrogels as smart, on demand drug delivery systems for the treatment of various NO-related diseases.

## Injectable RONS-releasing hydrogels

As RONS are generated as by-products of normal cellular metabolism, they are essential for cellular signaling at the physiological level. Therefore, RONS are ideal gaseous molecules that participate in cell signaling pathways to perform multiple biofunctions [80]. It is well known that ROS and NO exhibit broad-spectrum bactericidal activities against both gram-positive and gram-negative bacteria [81–83]. For example, H_2_O_2_, the most long-lived ROS, can eliminate bacteria through oxidative damage toward DNA, lipids and proteins, or through other mechanisms, including enzymatic assault, autophagy, mammalian target of rapamycin kinase inhibition and T-helper polarization [84]. Similarly, NO possesses antibacterial activities by directly covalently binding and cleaving DNA, lipids, and proteins at high concentrations. At low concentrations, NO also acts as an intracellular signal that regulates immune cells to clear pathogens [85, 86]. Due to this selective sensitivity of RONS to pathogens, many RONS-releasing hydrogels have been designed to combat infection, especially for infected wound treatment.

Another important application of RONS in biomedicine is in revascularization. Recently, an increasing number of studies have demonstrated that sufficient generation of NO can regulate the behavior of ECs and stimulate angiogenesis through several signaling pathways. Angiogenesis is a complex process involving the proliferation, differentiation and migration of ECs in the inner wall of blood vessels to induce the formation of new sprouts. Blood vessels (including arteries, arterioles, capillaries, venules and veins) are needed for all tissues and organs to supply and exchange oxygen and nutrients [87]. Therefore, moderating angiogenesis is an effective strategy for treatment of many cardiovascular diseases, such as deep vein thrombosis, blood clotting disorders, ischemic heart disease and coronary artery disease [88–94]. Angiogenesis plays a vital role in wound healing, taking part in the temporary formation of granulation tissue and supplying nutrients and oxygen to the growing tissue [74, 95]. It has been reported that exogenous ROS can stimulate the expression of angiogenic transcription factors (Ets-1, NF-kB and STAT-3) and other important genes (such as monocyte chemoattractant protein-1, vascular cell adhesion molecule 1 and MMPs), leading to the enhanced migration of ECs [96–104]. Specifically, sustained supply of H_2_O_2_ at low concentration (0.1–10 µM) was confirmed to encourage ECs proliferation, migration, and tube formation [96, 105]. Meanwhile, NO can induce a pro-angiogenic response by regulating the expression of crucial factors, such as vascular endothelial growth factor (VEGF), fibroblast growth factors, angiopoietin-2 and estrogen [104, 106].

The involvement of RONS in stem cell therapy has also been widely acknowledged. Stem cells are an essential building block in tissue regeneration that can differentiate into almost any type of cells that the body needs and can replace damaged cells after injury in degenerative diseases. Owing to their self-renewal, anti-inflammatory, immunomodulatory and signaling properties, stem cell transplantation is a promising therapeutic approach for many diseases, including diabetes, MI, cardiovascular diseases, chronic wounds and inflammatory diseases [107]. However, the low survival and retention rates of stem cells after transplantation to the defective sites remain significant challenges in stem cell-based therapy. In addition, emerging evidence indicates that within intracellular levels, RONS can modulate stem cell behaviors and adaption to the microenvironment, including viability, migration and differentiation [108, 109]. Therefore, RONS-releasing hydrogels are considered advanced carriers for improving the viability and functionality of encapsulated stem cells.

In the following section, we focus on the development of recent injectable hydrogels that can generate RONS, namely H_2_O_2_ and NO, at the cell signaling level, and discuss their potential applications in disinfection, vascular diseases and stem cell therapy.

### Injectable H_2_O_2_-releasing hydrogels

**Figure 8.**
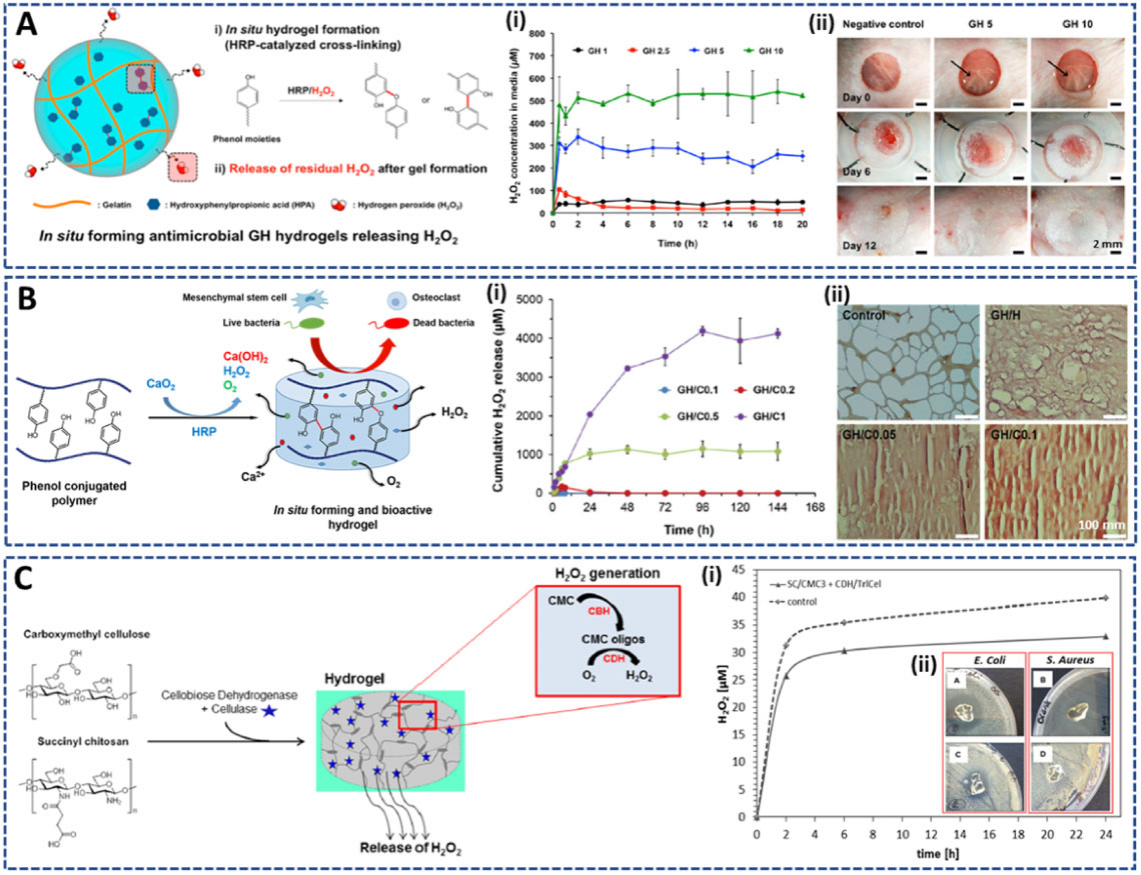
Injectable H_2_O_2_-releasing hydrogels for various applications. HRP-catalyzed crosslinked gelatin-based hydrogel for (**A**) loading high amount of H_2_O_2_ to treat infected wound: (**i**) H_2_O_2_ release amount from GH hydrogel, and (**ii**) *in vivo* wound healing efficiency. Reprinted and modified with permission from Ref. [119] Copyright 2017 American Chemical Society, and (**B**) for incorporation of CaO_2_ to enhance mesenchymal stem cell behaviors and antibacterial properties: (**i**) Cumulative H_2_O_2_ release, and (**ii**) stimulation effect on the differentiation of human MSCs. Reprinted and modified with permission from Ref. [120] Copyright 2020 the Royal Society of Chemistry. (**C**) Chitosan-based hydrogel prepared by carbodiimide chemistry for encapsulation of cellobiose dehydrogenase (CDH) and cellulases (TrlCel) to improve antibacterial activity: (**i**) cumulative generation of H_2_O_2_, and (**ii**) antibacterial effect toward *E. coli and S. aureus*. Reprinted and modified with permission from Ref. [121] Copyright 2017 American Chemical Society.

Horseradish peroxidase (HRP)-mediated crosslinking is a popular approach for hydrogel fabrication, in which H_2_O_2_ interacts with HRP to initiate the formation of radicals on phenol-containing polymers, resulting in crosslinking between phenols [110, 111]. Therefore, H_2_O_2_ plays a vital role in gelation and in controlling the physicochemical properties of hydrogels. Park *et al.* [55, 112–119] applied this method to develop various hydrogel systems for biomedical applications. In a previous study, they used H_2_O_2_ as the reduction substrate for hydrogel formation via an HRP-mediated reaction and to provide antibacterial activity (Fig. 8A) [119]. Interestingly, by varying the H_2_O_2_ feed amount (1–10 mM) during hydrogel fabrication, they controlled the release of H_2_O_2_ residues from the hydrogel in a wide range of 2–509 μM. Depending on the H_2_O_2_ release concentration, the hydrogel exhibited killing effects against both Gram-negative and Gram-positive bacteria, including the MRSA. Moreover, this hydrogel can promote the healing of full-thickness wounds in a rat model after 12 days and inhibit bacterial viability on the surface of infected scratched skin after 24 h. By applying an HRP-mediated crosslinking reaction, Park *et al.* [120] reported another novel method using CaO_2_ as a source of H_2_O_2_ to prepare dual H_2_O_2_/O_2_-releasing gelatin-based hydrogels (Fig. 8B). The decomposition of CaO_2_ provided H_2_O_2_ for the HRP-catalyzed crosslinking reaction, which gradually released H_2_O_2_ after hydrogelation. In addition, the decomposition of CaO_2_ in the hydrogel also generates other bioactive molecules, such as O_2_, and Ca^2+^ ions, which can improve the osteogenic differentiation of encapsulated stem cells. By varying the feeding amount of CaO_2_, the physico-chemical properties of hydrogels, such as gelation time, microporous structure, mechanical strength and release profile of bioactive molecules, can be well controlled. The combination of released H_2_O_2_, O_2_, and Ca^2+^ ions can stimulate the attachment, migration and differentiation of human MSCs, and exhibited antibacterial activity against both *Escherichia coli* and *Staphylococcus aureus*.

In addition to the direct encapsulation of H_2_O_2_ or H_2_O_2_ donors that release H_2_O_2_ during their decomposition, H_2_O_2_-releasing hydrogels can be prepared by incorporating enzymes that can catalyze the *in situ* generation of H_2_O_2_ through oxidation process. Guebitz *et al.* [121] developed a dual-enzyme catalyzed *in situ* H_2_O_2_ generating hydrogel, by utilizing the ability of cellobiose dehydrogenase (CDH) to produce H_2_O_2_ in the presence of cellobiose and molecular oxygen (Fig. 8C). For this approach, succinyl chitosan (SC) and carboxymethyl cellulose (CMC) were crosslinked through EDC/NHS chemistry to encapsulate CDH and cellulases (TrlCel) into the hydrogel. Thereafter, TrlCel hydrolyzed the crosslinked CMC in the SC/CMC hydrogel to produce oligomers as substrates for oxidization by CDH, which led to H_2_O_2_ release. The clinically relevant amount of H_2_O_2_ (30 µM for 24 h) from the hydrogel was able to inhibit bacterial (*E. coli* and *S. aureus*) growth and stimulate the proliferation of fibroblasts. Moreover, this H_2_O_2_ release system can be switched *on/off* by cooling in fridge conditions, providing a simple storage process. Although these H_2_O_2_-releasing hydrogels show excellent antibacterial effects on various bacterial strains without causing toxicity to mammalian cells, this study was limited to *in vitro* evaluations. More specialized studies in animal models are required to confirm the applicability of this hydrogel system in further medical practice.

**Figure 9.**
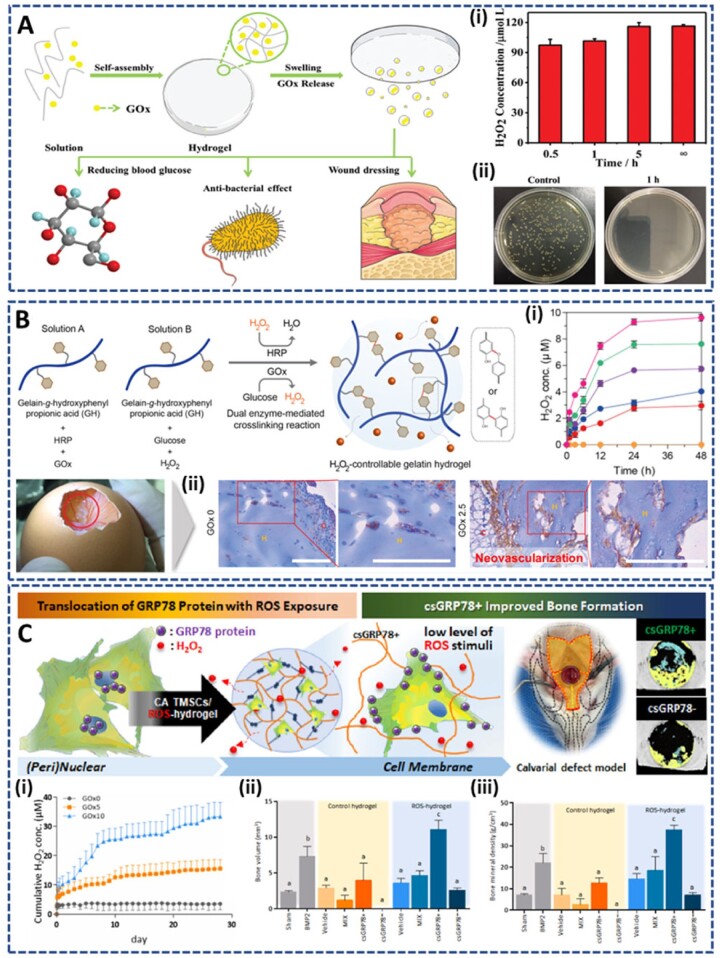
GOx-incorporated hydrogels releasing H_2_O_2_ in the presence of glucose: (**A**) antibacterial peptide-based prepared via self-assembly of IKYLSVN hetapeptide to reduce bacterial infection: (**i**) concentration of H_2_O_2_ released at different time, and (**ii**) excellent antibacterial effect against *E. coli.* Reprinted and modified with permission from Ref. [122] Copyright 2020 American Scientific Publishers. Gelatin-based hydrogel prepared by HRP-catalyzed crosslinking reaction to (**B**) accelerate the neovascularization: (**i**) cumulative release of H_2_O_2_ within 48 h, and (**ii**) neovascularization property through chorioallantoic membrane assay. Reprinted and modified with permission from Ref. [124] Copyright 2018 American Chemical Society, and to (**C**) promote bone regeneration by co-delivery of cell surface-specific GRP78 and tonsil-derived MSCs: (**i**) sustained release of H_2_O_2_ for upto 30 days, and improvement in (**ii**) bone volume and (**iii**) bone mineral density after implantation. Reprinted and modified with permission from Ref. [125] Copyright 2021 Elsevier Ltd.

Hyperglycemia in diabetic wounds is a favorable environment for bacterial growth, resulting in the unavoidable association of serious bacterial infections with the wound. Therefore, it is ideal to control the blood glucose level while treating bacterial infections in diabetic wounds. Glucose oxidase, GOx, is a popular enzyme that is widely used to catalyze the oxidation of glucose to generate H_2_O_2_ and gluconic acid. Taking advantage of this, Wang *et al.* [122] encapsulated GOx in the antimicrobial peptide hydrogel for both reducing blood glucose concentration while inhibiting the bacterial infection in diabetic wound (Fig. 9A). The hydrogel was prepared by self-assembly of the IKYLSVN heptapeptide (antibacterial peptide) via π–π stacking and hydrogen bonding. *In vitro* experiments demonstrated that the release rate of GOx could be well controlled by the swelling property of the hydrogel, leading to a significant reduction in the local glucose concentration and excellent antibacterial properties. This system is a promising dressing for promoting the healing of diabetic wounds.

Using GOx as a source for H_2_O_2_ generation, Park *et al.* [123] proposed a co-enzymatic hydrogelation system triggered by the combination of HRP and GOx. This HRP/GOx hydrogel system provides an artificial biocompatible microenvironment for a wide range of biomedical applications [123]. They applied this hydrogel to accelerate neovascularization (Fig. 9B) [124]. Herein, the addition of GOx can stimulate both the HRP-mediated crosslinking reaction and the release of H_2_O_2_ from the resultant hydrogels. The released amount of H_2_O_2_ (0–9.62 µM) was well controlled by varying the feeding concentration of GOx (0–5 μU/ml). The optimized H_2_O_2_ release amount (7.62 µM) from the GOx2.5 hydrogel temporally enhanced the initial intracellular ROS levels in human umbilical vein endothelial cell (HUVECs), leading to improved viability and angiogenesis. Moreover, *in vivo* experiments on chicken chorioallantoic membranes illustrated that acute oxidative stress induced by the burst release of H_2_O_2_ surrounding the tissue facilitated the neovascularization and tissue infiltration. This is a potential hydrogel system for clinical treatment of vascular-related diseases. However, further experiments are required to confirm the *in vivo* therapeutic effects of this system.

In another study, they continued to use HRP/GOx dual enzyme system to regulate the aging process of MSCs for enhancing the efficiency of MSC-based therapies (Fig. 9C) [125]. Glucose-regulated protein 78 (GRP78) is a specific senescence marker of MSCs, and the loss of GRP78 leads to age-related diseases. Based on these hypotheses, Park *et al.* encapsulated cell surface-specific GRP78 (csGRP78+) into the dual enzymatic crosslinking and H_2_O_2_-releasing hydrogel, to improve the quiescence and self-renewal of tonsil-derived mesenchymal stem cells (TMSCs). Notably, GOx5 is chosen as the optimal condition because of the appropriate H_2_O_2_ release amount (15.6 ± 0.1 μM), mechanical properties and biocompatibility for cell encapsulation. As expected, *in vitro* evaluations demonstrated an improvement in the regenerative capacity of TMSCs by regulation of GRP78, a unique MSC senescence marker, translocation and expression. Interestingly, implantation of csGRP78+ cells embedded in the H_2_O_2_-releasing hydrogel also promoted the new bone formation in a calvarial defect model. It is believed that this H_2_O_2_-releasing hydrogel is an advanced and promising injectable scaffold for MSC-based therapies.

### Injectable NO-releasing hydrogels

There are five main types of low-molecular-weight NO donors, including N-diazeniumdiolates (NONOates), S-nitrosothiols (RSNOs/SNOs), N-nitrosamines, nitrates and metal–nitrosyl complexes [6, 126]. Changes in physiological conditions (pH, light, temperature, moisture, etc.), enzymatic cleavage and reactions with metals or thiols can lead to the decomposition of LWM donors to release NO. These donors can be directly conjugated on the precursor polymer or embedded in the hydrogel matrix, and delivered to the target site. However, these approaches have some drawbacks, such as the shortage and instability of NO supply. Metals, metal ions and organoseleniums are popular agents that exhibit GPx-like catalytic activity for the decomposition of RSNO into NO. In addition, numerous endogenous RSNO species (e.g. S-nitrosoglutathione (GSNO), S-nitrosoalbumin (AlbSNO) and S-nitrosocysteine (CysNO)) coexist in fresh blood and can be a source of NO generation. Therefore, the delivery of more stable catalytic species (metals, metal ions, organoseleniums, etc.) to induce the sustained generation of NO from endogenous RSNO has received increasing attention [127, 128].

**Figure 10.**
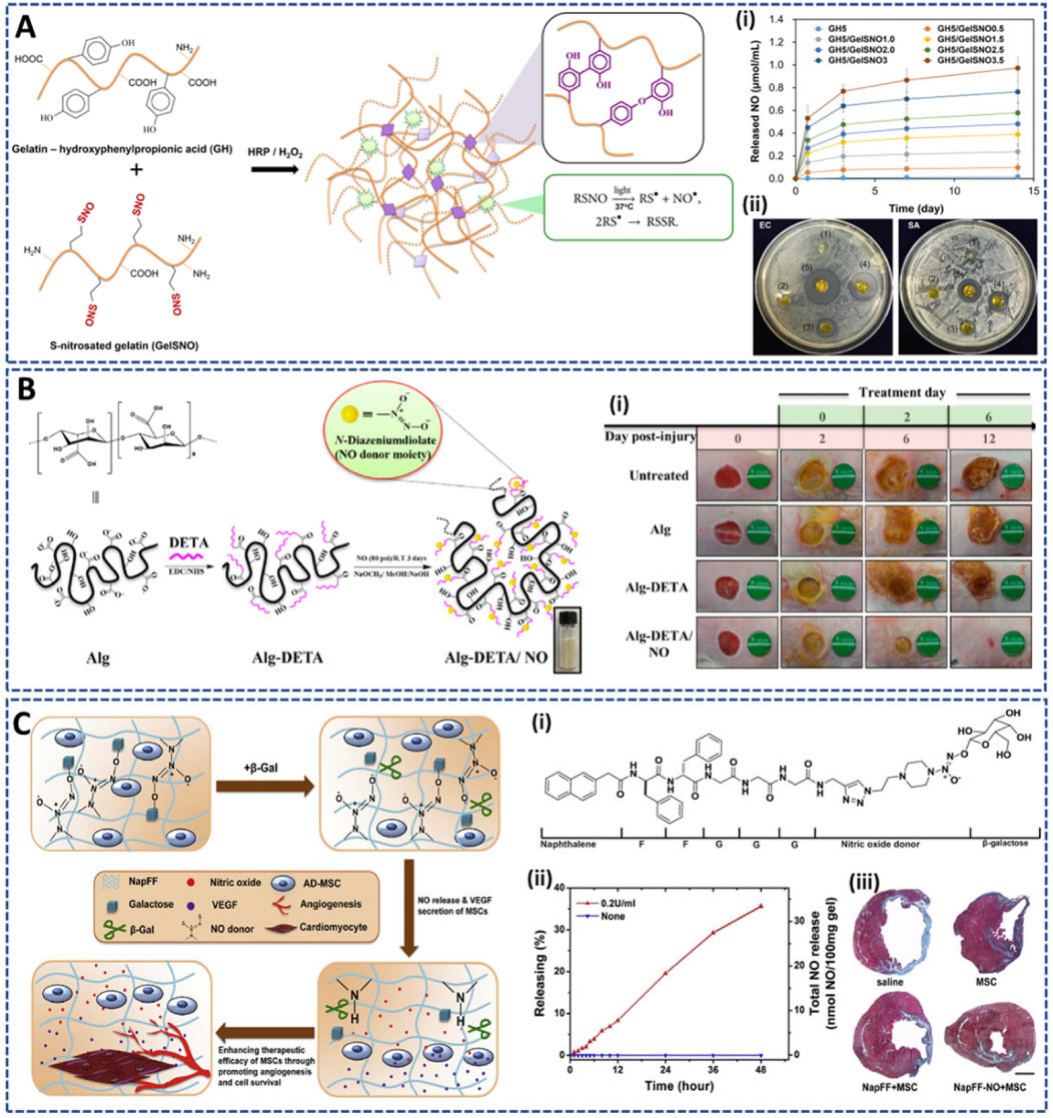
NO-releasing hydrogels prepared from NO donor-conjugated precursors: (**A**) S-nitrosothiolated gelatin hydrogel prepared via HRP-catalyzed reaction releasing NO by cleavage of S-N bonds under thermal and light excitation, to improve antibacterial property: (**i**) sustained release of NO from hydrogel for 14 days, and (**ii**) antibacterial effect of different hydrogel against *E. coli* and *S. aureus*. Reprinted and modified with permission from Ref. [113] Copyright 2018 Elsevier Ltd. (**B**) NONOate-doped alginate-based hydrogel crosslinked through carbodiimide chemistry releasing NO at 37°C to provide (**i**) antibacterial activity for infected wound treatment. Reprinted and modified with permission from Ref. [129] Copyright 2021 Elsevier Ltd. and (**C**) Naphthalene conjugated short peptide, FFGGG and b-galactose caged nitric oxide (NO) donor (NapFF-NO) self-assembled hydrogel releasing NO in response to b-galactosidase to enhance the therapeutic efficacy of mesenchymal stem cells for myocardial infarction: (**i**) structure of NapFF-NO, (**ii**) NO release amount and (**iii**) Masson’s trichrome staining image of heart sections at Day 30 after treated with hydrogel. Reprinted and modified with permission from Ref. [130] Copyright 2015 Elsevier Ltd.

Park *et al.* [113] developed an *in situ* forming NO-releasing hydrogel (GH/GelSNO) with excellent antibacterial properties (Fig. 10A). In this study, S-nitrosylated protein (SNO)-conjugated gelatin (GelSNO) was used as the NO source and was encapsulated in the HRP/H_2_O_2_-catalyzed crosslinked network of phenol-rich gelatin. Specifically, GelSNO was prepared through two-step synthesis, including conjugation of thiol groups on the gelatin backbone, and nitrosation of thiolated gelatin with NaNO_2_. Then, under several kinds of stimulations, such as visible light (330–350 and 550–600 nm), temperature or reduced thiols, the S-N bonds of GelSNO are cleaved to release NO. The amount of NO released from hydrogel (in PBS at 37°C) was varied from 0.72 to 15.14 nmol/ml for 4 days, by varying the content of GelSNO (0.5–3.5 wt%). Moreover, this hydrogel can generate peroxynitrite *in situ*, an active ingredient with stronger bactericidal properties than NO and H_2_O_2_, through the reaction between the released NO and residual H_2_O_2_. *In vitro* evaluations indicated the biocompatibility of the hydrogel with human dermal fibroblasts, while exhibiting remarkable antibacterial activities against both Gram-positive and Gram-negative bacteria. Therefore, this hydrogel is expected to be a promising material for infection treatment and tissue engineering in wound healing.

Yoo *et al*. [129] conjugated diethylenetriamine (DETA) onto the alginate (Alg) backbone and dopped in diazeniumdiolate (NONOate) to synthesize Alg-DETA/NO polymer powder, which has the NO content of around 0.23 μmol/mg polymer (Fig. 10B). Upon direct absorbance of the exudate, an Alg-DETA/NO hydrogel dressing can be formed at the wound site. The hydrogel exhibited the prolonged NO release for 4 days under physiological conditions (pH 7.4, 37°C), with ∼68% and 88% of NO released in the first 24 h and 48 h, respectively. The sustained release of NO from the hydrogel significantly inhibited the growth of MRSA, while increasing the number of fibroblasts. Moreover, *in vivo* experiments showed the promising effect of the Alg-DETA/NO hydrogel on eliminating infection and inflammation in MRSA-infected full-thickness wounds. However, different NO loaded contents should be investigated to demonstrate the various effects and potential of hydrogel systems in tissue regenerative medicine applications.

To improve heart remodeling after MI, Li *et al.* [130] prepared a ß-galactosidase-catalyzed NO-releasing hydrogel for transplantation of adipose-derived MSCs (AD-MSCs) (Fig. 10C). Some peptide sequences can self-assemble to form hydrogels via weak intramolecular or intermolecular interactions [122, 131–133]. Therefore, they used a naphthalene-modified-FFGGG short peptide sequence (NapFF) as a hydrogelator to offer better biocompatibility and biodegradability than common polymer gels. The NO-containing hydrogelator was then prepared through a click reaction between the alkyne of NapFF and the azide of a ß-galactose caged NO donor [134]. The hydrogel was then prepared by suspending NapFF and NapFF-NO with heat and cooling them back to room temperature, based on the self-assembly property of the peptide. In the presence of ß-galactosidase, 100 mg of NapFF-NO could sustainably release ∼33.32 nmol of NO for over 48 h. The co-transplantation of AD-MSCs and NO-releasing hydrogel increased the engraftment, survival and angiogenesis of MSCs, supporting heart remodeling and inducing neovascularization in MI mouse models.

In the above hydrogel system, the direct conjugation of the NO donor to the polymer precursor has some disadvantages, including instability of the functional groups due to heat, light and moisture during the synthesis and storage process. Therefore, the direct and simple loading of the NO donor into the hydrogel system is an alternative solution that eliminates the reagent loss during synthesis.

**Figure 11.**
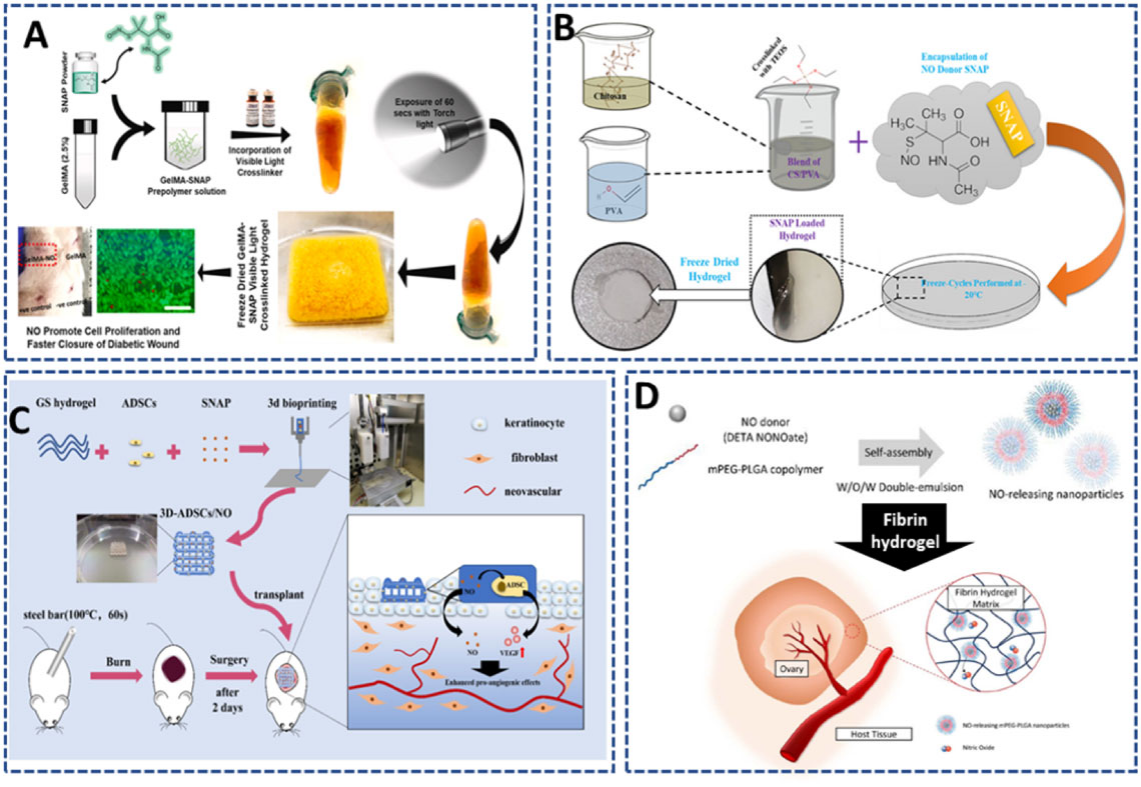
Low-molecular-weight NO donor-encapsulated hydrogel releasing NO under physiological conditions: (**A**) S-nitroso-N-acetyl-DL-penicillamine (SNAP)-loaded photocrosslinked gelatin-based hydrogel to accelerate infected wound closure. Reprinted and modified with permission from Ref. [135] Copyright 2021 Elsevier Ltd. (**B**) SNAP encapsulated chitosan-poly (vinyl alcohol) hydrogel crosslinked by tetraethoxysilane (TEOS) to promotes angiogenesis in chick embryo model. Reprinted and modified with permission from Ref. [136] Copyright 2019 Elsevier Ltd. (**C**) Co-delivery of adipose-derived mesenchymal stem cells and SNAP by 3D bioprinted gelatin-based hydrogel to promote severe burn wound healing. Reprinted and modified with permission from Ref. [137] Copyright 2021 Oxford University Press. (**D**) Encapsulation of diethylenetriamine NONOate (DETA NONOate)-loaded methoxy polyethylene glycol-(poly(lactic-co-glycolic acid)) (mPEG-PLGA) nanoparticles in fibrin hydrogel to coat the ovaries for promotion of angiogenesis toward transplanted ovaries. Reprinted and modified with permission from Ref. [138] Copyright 2021 IOP Publishing.

Hasan *et al.* [135] designed an S-Nitroso-N-acetylpenicillamine (SNAP) loaded visible light crosslinked gelatin methacryloyl (GelMA) hydrogel patch for diabetic wound (Fig. 11A). The GelMA/SNAP hydrogel was prepared simply by adding SNAP at different concentrations into the GelMA precursor and photocrosslinking in the presence of an initiator and normal torchlight (410–440 nm) for 60 s. GelMA/SNAP showed a prolonged and sustained NO release up to 80 h, which is controllable and depends on the feeding amount of SNAP. By choosing suitable conditions, this hydrogel can enhance the proliferation and migration rates of fibroblasts and keratinocytes, leading to rapid wound closure. Moreover, with the sustained NO-releasing capacity, GelMA/SNAP can stimulate 100% wound contraction in a streptozotocin-induced type 1 diabetic rat model through the higher re-epithelialization and complete formation of the stratum corneum.

In another study, this group developed a chitosan-poly (vinyl alcohol) hydrogel that can release NO to accelerate angiogenesis in the chick embryo model (Fig. 11B) [136]. The hydrogel was crosslinked with chitosan (CS) and poly (vinyl alcohol) (PVA) using non-toxic tetraethoxysilane (TEOS). SNAP (5‰ and 10‰) was used as an NO donor and loaded into the hydrogel at different concentrations. The accumulation of SNAP on the surface of the hydrogel led to an early burst release within 3 h. Subsequently, NO was released more slowly and steadily over time, which was associated with the swelling and degradation rate of the hydrogel. The cumulative NO release amount was over 100 µM after 120 h. The burst release can reduce the attachment and proliferation of proteins, platelets and bacteria at an early stage, while the sustained release can promote endothelization later. The CS-PVA hydrogel loaded with 5‰ SNAP stimulated faster wound closure during *in vitro* studies using keratinocytes and fibroblast cells. Meanwhile, CS-PVA containing 10‰ SNAP showed a slightly better effect on inducing the formation of new blood vessel in the chick embryo model, indicating the usefulness of the CS-PVA-SNAP hydrogel in enhancing angiogenesis. Nevertheless, additional studies are required to understand better the mechanisms and effects of this system in clinical applications.

In addition to many studies on MI treatment, AD-MSCs transplantation is also a potential strategy for wound healing therapy. To promote severe burn wound healing, Zhu *et al.* [137] designed a three-dimensional (3D) bioscaffold prepared from gelatin-based hydrogel for co-encapsulation of AD-MSCs and NO donors (Fig. 11C). Specifically, a gelatine-sodium (GS) alginate hydrogel containing adipose-derived stem cells (ADSCs) suspension and SNAP was extruded through a bioprinter to form a square grid scaffold and crosslinked by immersing in a calcium chloride solution. The resulting 3D-ADSC/NO scaffold exhibited sustained NO release for up to 5 days with the burst within the first 4 h. In addition, the encapsulated SNAP did not inhibit the proliferation of ADSCs. Moreover, 3D-ADSC/NO constructs stimulated both the *in vitro* and *in vivo* migration and angiogenesis of HUVECs, which may be associated with the secretion of VEGF. *In vivo* burn wound healing experiments also confirmed the benefits of scaffolds in inducing collagen deposition, rapid epithelialization and neovascularization by participating in the VEGF signaling pathway. It is also illustrated that the ability of ADSCs in the 3D-ADSC/NO scaffold to secrete cytokines and VEGF may be associated with their angiogenic properties. These findings suggest that the 3D-ADSC/NO hydrogel can be a novel therapeutic approach for severe burn wound healing.

Interestingly, NO-releasing hydrogels can also be used to promote angiogenesis in transplanted ovaries to improve fertility in cancer survivors. Cryopreservation of ovarian tissue is a favored fertility preservation method; however, the recovery of ovarian function after freeze-thawing remains an obstacle. To improve graft survival and function, the ovaries were coated with fibrin hydrogel containing NO-releasing nanoparticles (NO-NPs) (Fig. 11D). [138]. First, NO-NPs were synthesized via the conventional double-emulsion method, using a methoxy polyethylene glycol-(poly(lactic-co-glycolic acid)) copolymer (mPEG-PLGA) and diethylenetriamine NONOate (DETA NONOate). The fibrin hydrogel was then introduced with NO-NPs and coated on the ovaries before transplantation. The amount of NO released from the NO-NPs was sustained at a low level for 48 h, even after loading in the fibrin hydrogel. Coating with NO-releasing fibrin hydrogels significantly improved the vascularity of the transplanted ovaries by 4.78 folds, determined by an increase in CD31 expression. *In vitro* experiments confirmed that fertilizable oocytes could develop into normal blastocysts, indicating their function as reproductive organs. Moreover, the formation of new blood vessels on the transplanted ovaries inhibited the loss of follicles and prevented ischemic damage The results obtained from this study reveal the potential of NO-releasing hydrogels for use in various biomedical applications, in which angiogenesis is a necessary factor.

**Figure 12.**
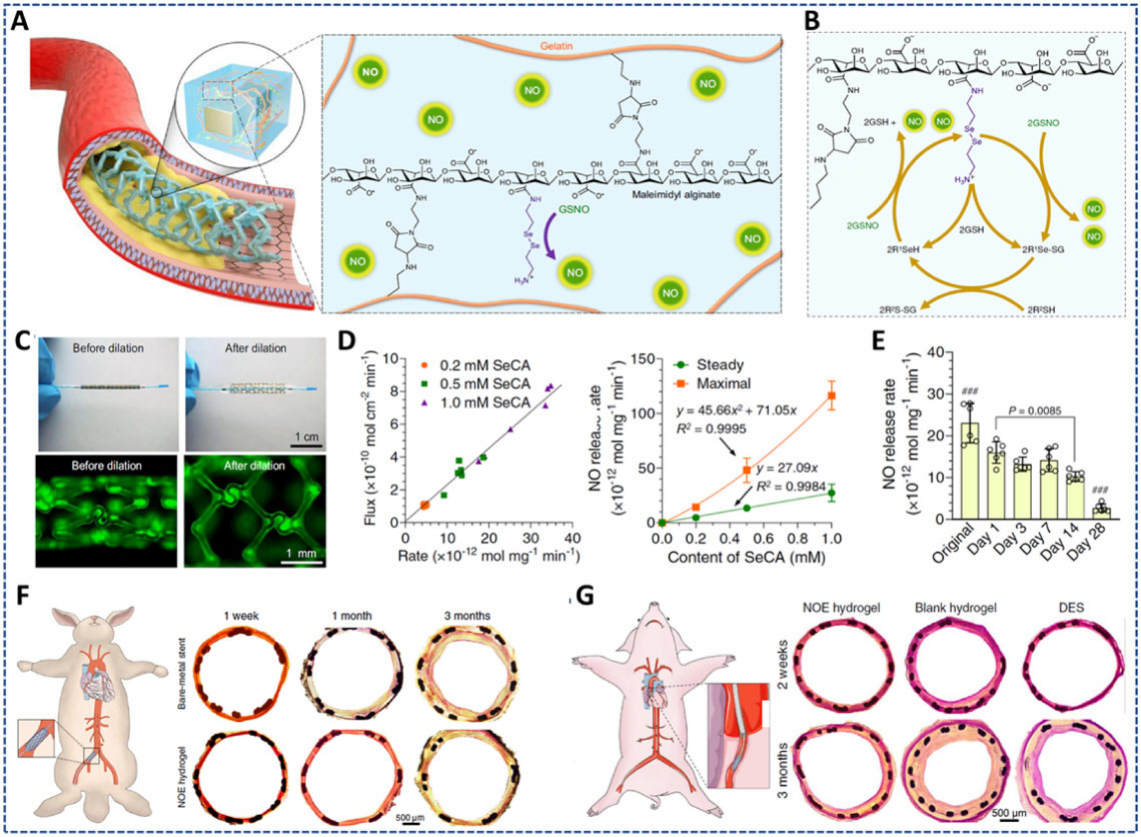
Development of mechanically tough NO-eluting (NOE) hydrogel for coating vascular stent. (**A**) Selenocysteine (SeCA)-conjugated maleimide-alginate polymer and gelatin polymer were crosslinked via Michael addition to prepare tough hydrogel for vascular stent coating. (**B**) SeCA on the coated stent interact with endogenous S-nitrosothiol to generate NO. (**C**) Vascular stent coated with hydrogel before and after balloon dilation. (**D**) Correlation between the fluxes and rates of NO generation. (**E**) Release rate of NO after preincubated in PBS. (**F**) Vascular stent deployment in rabbits. (**G**) Vascular stent deployment in pigs. Reprinted and modified with permission from Ref. [140] Copyright 2021 Nature Communications.

The implantation of vascular stents and self-expanding metal mesh tubes in the coronary artery to open a blocked blood vessel and improve blood flow is a popular approach in the treatment of cardiovascular diseases [139]. However, the thickness of the vascular wall and loss of luminal patency, known as neointimal hyperplasia, usually leads to implantation failure. At that time, Wu *et al.* [140] designed a tough NO-eluting (NOE) hydrogel for coating a vascular stent to suppress neointimal hyperplasia (Fig. 12). To form a mechanically tough hybrid hydrogel, maleimide-modified alginate and gelatin were crosslinked via Michael addition. It was confirmed that both physical interactions within gelatin hydrogel (below its melting point) [141] and chemical crosslinking between the amine and maleimide groups contributed to the mechanical strength of the hydrogel. In this study, selenocysteine (SeCA) was used as an NO donor and was conjugated to maleimide-alginate. SeCA can catalyze the generation of NO from S-nitrosothiols, which is activated by the interchange reaction of diselenide de(R’Se-SeR’) and thiol (R-SH) [142]. In the presence of GSNO, the NOE hydrogel stimulated the burst release of NO for a few minutes before reaching the steady state, depending on the content of SeCA. The NOE hydrogel also showed stability in catalytic activity after preincubation in PBS for up to 2 weeks. Consequently, the NOE-coated surfaces selectively enhanced the adhesion of ECs, while inhibiting the development of smooth muscle cells due to the biological effect of NO. NOE hydrogel-coated stents not only persistently suppressed inflammation and neointimal hyperplasia but also promoted the rapid restoration of the native endothelium in both leporine and swine models. Based on these results, it is believed that this NOE hydrogel is a promising material for coating various vascular implants. In addition to healthy animal models, the biological activity of this system should be evaluated in pathological animal models.

In addition to the typical NO donors described above, many new donors have been designed to target a more stable and specific release of NO. These donors release NO only under specific stimulation or pathological conditions [127]. Moreover, to increase the biological efficiency or provide multiple functions to hydrogels, recent concepts tend to combine the activities of two or more biological molecules or strategies.

**Figure 13.**
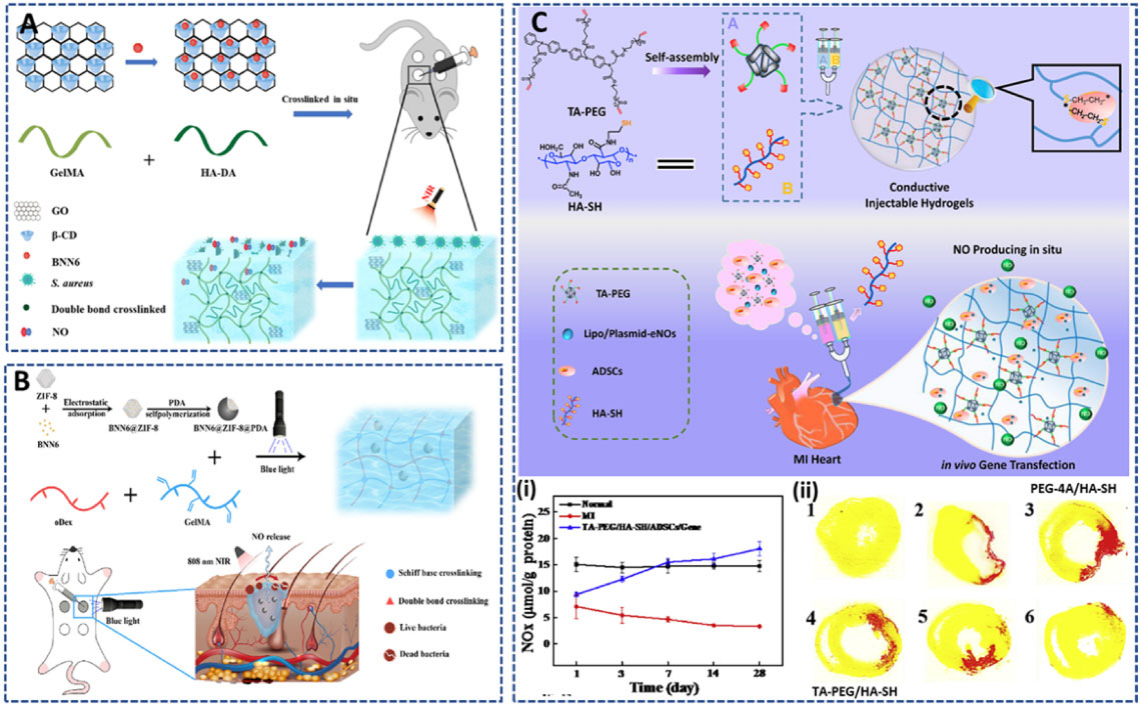
Near-infrared (NIR)-triggered-crosslinked hydrogel with synergistic antibacterial properties from photothermal therapy (PTT) and NO for infected wound treatment: (**A**) β-cyclodextrin βCD-functionalized GO (GO-βCD) nanoparticles incorporated in methacrylate-modified gelatin/hyaluronic acid-grafted dopamine (GelMA/HA-DA) hydrogel for delivery of Bis-N-nitroso compounds (BNN6). NO was released from BNN6 under irradiation via NIR laser. Reprinted and modified with permission from Ref. [145] Copyright 2020 American Chemical Society. (**B**) Zinc-methylimidazolate framework-8 particles (ZIF-8) modified polydopamine (PDA) (BNN6@ZIF-8@PDA) nanoparticles encapsulated in a gelatin methacrylate (GelMA)/oxide dextran (oDex) hydrogel for delivery of BNN6. Reprinted and modified with permission from Ref. [154] Copyright 2020 Elsevier Ltd. (**C**) Tetraaniline-polyethylene glycol diacrylate (TA-PEG) nanoparticles encapsulated thiolated hyaluronic acid (HA-SH) hydrogel for delivery of lipo/plasmid DNA-encoding eNOS (DNA-eNOS) nanocomplexes and AD-MSCs conductive hydrogel to target the treatment of myocardial infraction: (**i**) NOx concentration in a 4 week time period in normal and MI hearts, and in the MI hearts with TA-PEG/HA-SH/ADSCs/gene hydrogel treatment, and (**ii**) sirius red staining of left ventricle at 28 days after different treatments. Reprinted and modified with permission from Ref. [155] Copyright 2018 Elsevier Ltd.

Besides the controllable delivery of antibacterial agents, photothermal therapy (PPT) is another common method to fabricate antibacterial hydrogels for wound dressings. This method utilizes the excitation of a photosensitizer (gold, graphene, dopamine) [143–147] under a specific light irritation to produce heat, which kills the target cells. PPT is considered as a potentially effective technique for targeting the treatment of bacterial infections due to its minimal invasiveness, high penetration and reduced side effects [143, 148–153]. Therefore, Guo *et al.* [145, 154] combined NO and PPT therapies to synergistically treat bacteria-infected wounds. In their studies, Bis-N-nitroso compounds (BNN6) were used as NO donors, which can release NO under irradiation via a near-infrared (NIR) laser (UV light, 365 nm). To increase the adsorption and solubility of BNN6 into hydrogel, β-cyclodextrin (βCD) and hydrophobic zinc-methylimidazolate framework-8 particles (ZIF-8) were used as the effective carries for BNN6 delivery. In addition, they chose GO and polydopamine (DA) as the photosensitizer to exert the PPT in these studies, respectively. In the previous study, βCD-functionalized GO (GO-βCD) nanoparticles were incorporated into a methacrylate-modified gelatin/hyaluronic acid-grafted dopamine (GelMA/HA-DA) precursor to prepare an NIR (420 nm)-triggered crosslinked hydrogel (Fig. 13A) [145]. This hydrogel exhibited the enhanced adhesive properties owing to the HA-DA component. The loading amount of BNN6 by GO-βCD was higher than that of GO alone, leading to a higher release of NO under NIR irradiation (880 nm, 2 W/cm^2^). The NO release reached a value of 4 µM after 20 min under NIR irradiation. GO-βCD also exhibited high photothermal conservation and stability, reaching over 50°C and repeating after more than four cycles. In a subsequent study, ZIF-8 carrying BNN6 was modified with polydopamine (PDA) to prepare BNN6@ZIF-8@PDA nanoparticles, which reduced the toxicity and improved the photothermal activities of BNN6. The nanoparticles were then encapsulated in a gelatin methacrylate (GelMA)/oxide dextran (oDex) hydrogel (Fig. 13B) [154]. The final hydrogel product was fabricated through Schiff base crosslinking and photocrosslinking (blue light, 405 nm). Under irradiation with an 808 nm (2 W/cm^2^) NIR laser, PDA exhibited excellent photothermal-conversion, and BNN6 was photochemically degraded to release NO (7 µM after 3 h). In both systems, the synergetic effect of heat (over 50°C) and released NO significantly improved the bactericidal effect against *S. aureus* and *E. coli*, compared with the hydrogel without NIR irradiation. These combined hydrogel systems also inhibited inflammation, enhanced angiogenesis and promoted collagen deposition, resulting in the regeneration of sebaceous glands and complete healing of full-thickness defects in a rat model. Overall, the synergistic effects of PPT and NO can remarkably improve the antibacterial efficiency and reduce drug resistance for wound healing applications.

Currently, novel strategies using conductive biomaterials that induce a good cellular response and promote cell–cell signaling transduction, have been reported to bring many advantages to cardiac tissue engineering. Jia *et al.* [155] developed an injectable, conductive hydrogel for the co-delivery of AD-MSCs and plasmid DNA-encoding eNOS to target the treatment of MI (Fig. 13C). To endow hydrogels with conductive activity, tetraaniline (TA), an excellent electroactive material similar to polyaniline, was conjugated with polyethylene glycol diacrylate (PEG) [156]. Tetraaniline-polyethylene glycol diacrylate (TA-PEG) can self-assemble via hydrophobic and π–π interactions to form nanoparticles. Thereafter, the conductive hydrogel was prepared from the click reaction between the acrylate groups in TA-PEG and thiol groups in thiolated hyaluronic acid (HA-SH). Lipo/plasmid DNA-encoding eNOS (DNA-eNOS) nanocomplexes and AD-MSCs were also encapsulated inside this conductive hydrogel to ensure their closeness for supporting interactions. As mentioned previously, eNOS is among the three endogenous sources of NO synthesis in ECs. Therefore, delivery of DNA-eNOS is an effective way to improve the endogenous generation and bioavailability of NO. The electrical conductivity of the TA-PEG/HA-SH hydrogel was dependent on the content of TA-PEG, which achieved a conductivity of 2.32 × 10^−4^ S/cm (within the range of native myocardium conductivity) when 7.5% TA-PEG was used. The achieved conductivity improved the electrical signal between adjacent cardiac cells and restored the lost electrical conductivity in the scarred peri-infarct region. Direct injection of hydrogel into the infarcted myocardium eliminated the inflammatory response and reduced number of AD-MSCs. In addition, the sustained release of NO within 4 weeks stimulates the remarkably stronger expression of eNOS, Cx43, a-Actinin, alpha smooth muscle actin (a-SMA) and Von Willebrand Factor (vWF) in the myocardium, indicating the clear formation of new blood vessels and regeneration of themyocardium. This multifunctional hydrogel design represents an optimal therapeutic strategy for the treatment of MI.

## Conclusions and perspectives

RONS-controlled biomaterials have been proposed as potential therapeutics for the dual roles of RONS in physiological and pathological conditions. In particular, injectable hydrogels are representative of innovative biomaterials owing to their 3D and ECM-mimicking structures, minimal invasiveness and easy encapsulation of therapeutic agents, including biomacromolecules, cells and small molecules. In addition, they are easily modified for multifunctionalities, such as electroconductivity, self-healing, homeostasis, adhesiveness and stimuli responsiveness for specific applications. Therefore, they are believed to be successful and promising biomaterials for use as scaffolds and carriers for delivering/scavenging RONS for tissue regeneration and disease therapies.

Although many injectable RONS-controlling hydrogels have been developed for *in vitro*/*in vivo* evaluations, their clinical applications remain challenges, which is associated with the safety concern and stability of the materials, as well as the high variations in ROS expression between patients and disease conditions. Also, majority of RONS-generating hydrogels have not been thoroughly evaluated *in vivo*. Therefore, there are critical points that need to be carefully considered for further clinical applications. For example, in the case of ROS-scavenging hydrogels, it is dangerous if ROS are suppressed to below the normal physiological level. In contrast, the burst and high ROS concentrations released from ROS-generating hydrogels may induce toxicity in healthy organs/tissues. Thus, the mechanism and sufficient ROS scavenging/producing amount for redox balance and biological safety need to be precisely controlled and optimized, depending on the specific conditions of the target diseases. In addition, the toxicity of ROS scavengers, especially inorganic nanoparticles embedded within the hydrogel matrices, as well as the long-term stability and degradability of hydrogels, are needed for further investigation under specific physiological conditions in which hydrogels are supposed to be applied. In case of RONS-triggering drug release, the RONS-sensitive linkers incorporated in the hydrogels should be stable at very low ROS levels, but effectively release the active agents as the ROS levels increases at the pathological sites. It is noted that the intrinsic ROS level in tumors is relatively low, other ROS-generating agents should be incorporated in the hydrogels to increase the tumoral ROS levels. Lastly but importantly, the appropriate physicochemical properties, such as gelation rate, mechanical strength and adhesive strength of hydrogels, which may affect the ROS scavenging and drug release behaviors, also need to be considered when designing hydrogel for real applications.

In conclusion, injectable RONS-controlling hydrogels have been showing the therapeutic potential in regenerative medicine and tissue regeneration. In combination with other inherent properties of synthetic, natural or hybrid/nanocomposite hydrogels, such as self-healing, adhesiveness, conductivity and stimuli responsive for drug delivery, these RONS-controlling hydrogels are expected to be advanced, multifunctional platforms to enhance the treatment of various RONS-related diseases.

## Funding

This work was supported by a grant from Priority Research Centers Program (2019R1A6A1A11051471) funded by the National Research Foundation of Korea (NRF) and Korea Medical Device Development Fund grant funded by the Korea government (the Ministry of Science and ICT, the Ministry of Trade, Industry and Energy, the Ministry of Health & Welfare and the Ministry of Food and Drug Safety) (Project Number: RS-2020-KD000033), and Korea Evaluation Institute of Industrial Technology (KEIT 20018560, NTIS 1415180625) funded by the Ministry of Trade, Industry & Energy (MOTIE, Korea).

*Conflicts of interest statement*. The authors declare no conflict of interest.
